# Optical synaptic devices with ultra-low power consumption for neuromorphic computing

**DOI:** 10.1038/s41377-022-01031-z

**Published:** 2022-11-29

**Authors:** Chenguang Zhu, Huawei Liu, Wenqiang Wang, Li Xiang, Jie Jiang, Qin Shuai, Xin Yang, Tian Zhang, Biyuan Zheng, Hui Wang, Dong Li, Anlian Pan

**Affiliations:** 1 Key Laboratory for Micro-Nano Physics and Technology of Hunan Province, State Key Laboratory of Chemo/Biosensing and Chemometrics, College of Materials Science and Engineering, Hunan University, 410082 Changsha, China; 2 Hunan Institute of Optoelectronic Integration, Hunan University, 410082 Changsha, China; 3 School of Physics and Electronics, Central South University, 410083 Changsha, China

**Keywords:** Photonic devices, Optoelectronic devices and components

## Abstract

Brain-inspired neuromorphic computing, featured by parallel computing, is considered as one of the most energy-efficient and time-saving architectures for massive data computing. However, photonic synapse, one of the key components, is still suffering high power consumption, potentially limiting its applications in artificial neural system. In this study, we present a BP/CdS heterostructure-based artificial photonic synapse with ultra-low power consumption. The device shows remarkable negative light response with maximum responsivity up to 4.1 × 10^8^ A W^−1^ at *V*_D_ = 0.5 V and light power intensity of 0.16 μW cm^−2^ (1.78 × 10^8^ A W^−1^ on average), which further enables artificial synaptic applications with average power consumption as low as 4.78 fJ for each training process, representing the lowest among the reported results. Finally, a fully-connected optoelectronic neural network (FONN) is simulated with maximum image recognition accuracy up to 94.1%. This study provides new concept towards the designing of energy-efficient artificial photonic synapse and shows great potential in high-performance neuromorphic vision systems.

## Abstract (web summary)

Energy-efficient artificial photonic synapse is designed based on photo-sensitive BP/CdS heterostructure device, which can be used for high-performance brain-inspired neuromorphic computing.

## Graphical abstract

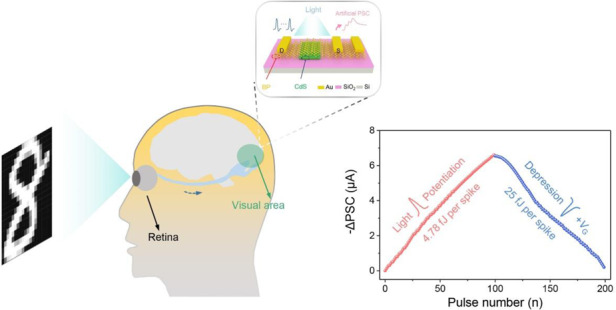

## Introduction

Artificial intelligence (AI), as a new branch of computer science, is seeking to understand the nature of intelligence and aiming to perform complex tasks that would normally require human intelligence^1^. Since the beginning of this century, with the rise of internet big data, the explosive growth of information, AI system has entered a new period of rapid development and gains more and more attention^2–4^. Rather than traditional von Neumann architecture-based computing system, the newly emerged brain-inspired neuromorphic computing is featured by parallel computing that reacts in a manner similar to human brain and thus possesses high efficiency and low power consumption^5,6^. It can be imagined that the future scientific and technological products brought by neuromorphic computing will be the “container” of human wisdom.

Similar to synapses in human brain, artificial synapses are considered as core components in constructing brain-inspired neuromorphic computing and play significant role in transmitting signals between synaptic neurons^7–9^. Up to now, different artificial synapse prototypes have been successfully constructed based on organic materials^10–12^, perovskites^13,14^ and low-dimensional materials^15,16^. Most of the reported works are focused on electrically stimulated synapses that are trained by electrical signals and thus endowed with learning and cognitive functions^5,9,17,18^. For example, Kinam Kim^18^, the vice chairman and CEO of Samsung Electronics, has teamed up with scientists at Harvard University to come up with a vision that promises real human brain function: Directly copy the brain’s neural signal and interconnection mode, and paste it on the electronic computer framework, using electronic current instead of biological current signal, so as to realize a real artificial neural network. In addition to electric-stimulated synaptic devices, optical-stimulated synapses have also been seriously considered ascribing to the advantage of high bandwidth, fast speed, and low cross-talk characteristics^4,10–16,19–22^. More importantly, such photonic synapses can simulate typical synaptic plasticity behavior under optical stimulation, which is beneficial to the development of artificial vision system. For example, Zhou et al.^19^ demonstrated two-terminal optoelectronic resistive random access memory (ORRAM) synaptic device with a structure of Pd/MoO_x_/ITO, which exhibits UV-light-tunable synaptic behaviors. The results show that the ORRAM array not only allows us to perform a first-stage image processing, but also effectively improves the processing efficiencies and accuracy of subsequent processing tasks. However, due to the large power of the stimulating optical signal, the reported photonic synapses are still far from practical applications. In this term, to develop artificial photonic synapse with low power optical plasticity and high image recognition accuracy is of great significance.

In this work, we present an artificial photonic synapse based on BP/CdS van der Waals heterojunction, where the CdS and BP are employed as the photosensitive layer and channel layer, respectively. Basic photoresponse behavior is probed with a laser source of 450 nm, where record high responsivity of 4.1 × 10^8^ A W^−1^ can be deduced with incident light power of 0.16 μW cm^−2^. Such sensitive photoresponse enables the applications of the device as synapse with ultra-low average power optical plasticity ~4.78 fJ per spike. Based on typical optoelectronic synaptic behavior of the artificial photonic synapse, a fully-connected optoelectronic neural network (FONN) is further constructed to evaluate the accuracy of image recognition for the Modified National Institute of Standards and Technology (MNIST) handwriting image dataset. The results show that maximum recognition accuracy of 94.1% can be achieved after training. This work provides a new strategy for the design and fabrication of energy-efficient artificial photonic synapses for constructing high-performance neuromorphic computing systems.

## Results

### Biological synapse and the designed artificial synaptic device

**Fig. 1:**
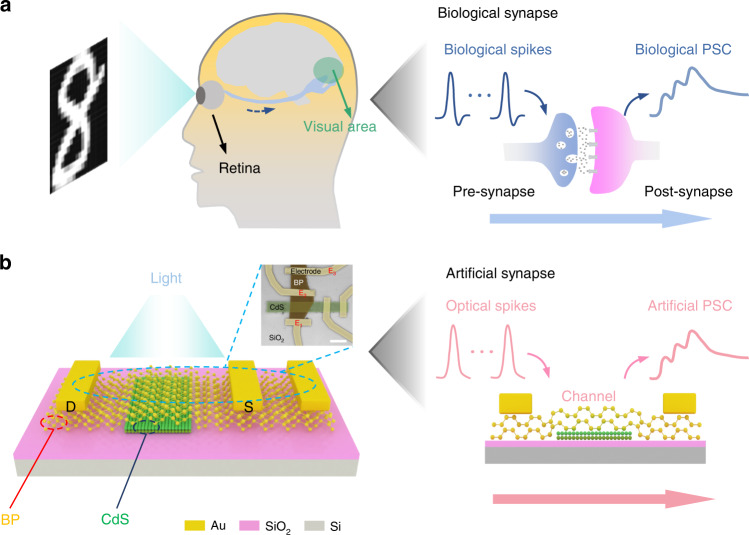
Comparison of the biological synapse with the designed artificial synaptic device. **a** Schematic illustration of human visual sensory system. The right panel of figure describe the responses to biological spikes in the biological synapse. **b** Schematic structure of the artificial photonic synapse based on BP/CdS vdWs heterojunction. Upper panel: top-view scanning electron microscope (SEM) image of the device; scale bar, 5 μm. The light green strip is CdS, while the brown area indicates the BP flake. The right panel of figure describe the responses to optical spikes in the artificial synapse, Cr/Au (10 nm/50 nm) electrodes are only contact with BP

Figure 1a depicts the schematics of the human visual system, where the information detected by eyes is further passed through optic nerves and processed in the visual area in the brain. The synapses are the connection between each neuron and play crucial role in neural information transmission. As shown in the right panel of Fig. 1a, when the presynaptic terminal is stimulated by biological spikes, neurotransmitters are released by presynaptic membrane and transmitted to the receptors of the postsynaptic terminal, leading to the variation of the amplitude of postsynaptic current (PSC). In this sense, designing artificial synapse with optical plasticity is critically important to enable the machine vision and neuromorphic computing. Figure 1b shows the schematic illustration of the designed artificial photonic synapse in this work. It is based on a BP/CdS heterostructure transistor, where the CdS flake obtained by chemical vapor deposition is imbedded under an exfoliated multi-layer BP as photogating and charge trapping layer. Raman and PL measurement is conducted to characterize each component, where the acquired Raman spectrum of BP and PL spectrum of CdS are shown in Fig. S1c and Fig. S1d, respectively. Inset of Fig. 1b presents a false-color scanning electron microscope (SEM) image of a designed device. The corresponding optical image and atomic force microscopy (AFM) image are shown in Fig. S1a and Fig. S1b, where the thickness of the BP and CdS is identified to be 16 nm and 33 nm, respectively. Simplified working process of the artificial synapse is schematically illustrated in the right panel of Fig. 1b, where optical spikes are used as trigger thus to drive the synaptic device.

### Optoelectronic characteristics

**Fig. 2:**
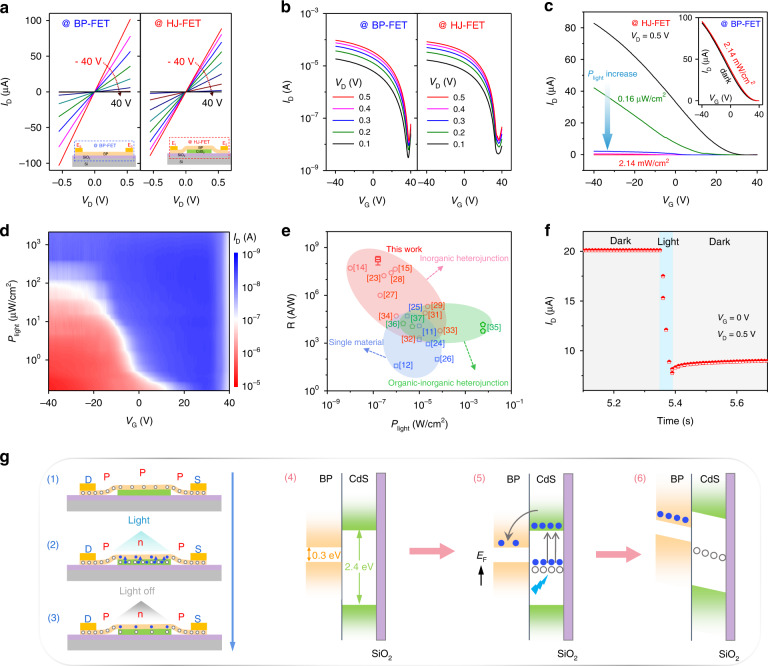
Optoelectronic characteristics of the designed device and corresponding working mechanism. **a** The output characteristics of HJ-FET and BP-FET at various back gate voltage under dark condition. Inset: the front diagram of vdWs optoelectronic synaptic device, device between electrodes E_1_ and E_2_ is named as heterojunction FET (HJ-FET), device between electrodes E_2_ and E_3_ is named as normal BP FET (BP-FET). **b** The transfer curves of HJ-FET and BP-FET at different *V*_D_ under dark condition, both showing a strong p-type and weak n-type behavior. **c** The transfer curves of HJ-FET in dark condition and different light power illumination. *V*_D_ is fixed at 0.5 V, showing an obvious negative photoresponse. **d** 2D plot of *I*_D_ as a function of light power intensity (*P*_light_) and *V*_G_. **e** R summary of photoelectronic device based on single and hybrid materials, demonstrating an ultra-high responsivity among reported devices. **f** Photoresponse current of the artificial synapse at *P*_light_ = 11.97 μW cm^−2^ and *V*_G_ = 0 V with light pulse width at 50 ms. **g** Charge-trapping state and corresponding energy band diagram of the HJ-FET in dark condition (g_1_ and g_4_), under light illumination (g_2_ and g_5_) and after removing light source (g_3_ and g_6_). *E*_F_: Fermi level

Basic transport and photoresponse properties of the fabricated devices were firstly probed in a vacuum chamber of ~10^−4^ to eliminate the effects of oxygen and water in the air. Figure 2a shows the measured output curves at different gate voltages applied on Si (300 nm SiO_2_ used as insulating layer), where symmetric and linear relationship can be observed in both heterojunction FET (HJ-FET, E_1_ and E_2_) and normal BP FET (BP-FET, E_2_ and E_3_). Meanwhile, similar ambipolar transfer characteristics are also acquired at the HJ-FET and BP-FET (Fig. 2b), indicating that the imbedding of CdS flake has no obvious influence on the electrical properties of BP at dark condition. The comparison on the maximum current (*I*_max_), on-off ratio and mobility shown in Fig. S2 also confirm the results. However, when the device is applied with light illumination, different transport behaviors are observed. Transfer behavior of BP-FET is depicted in inset of Fig. 2c, which shows normal and weak positive light response towards laser irradiation^23^. On the contrary, the HJ-FET shows distinct negative photoresponse (Fig. 2c). Figure 2d is a two-dimensional plot of *I*_D_ as a function of *P*_light_ and *V*_G_, showing the negative optical response information of the HJ-FET in more detail. It can be inferred from Fig. 2d that the drain current dramatically decreases with the *P*_light_ increasing from 0.16 μW cm^−2^ to 2.14 mW cm^−2^. We also probed the photoresponse behavior of pure CdS as a comparison (Fig. S3). The CdS FET also shows normal positive photoresponse, indicating that the negative photoresponse observed in the heterostructure originates from the heterointerface rather than the conduction variation in each component.

The novel transport behavior in BP/CdS heterostructure can be reasonably understood by the working mechanism shown in Fig. 2g. In dark condition, because the electrodes are only in direct contact with BP, the HJ-FET exhibits similar electrical properties as compared with BP-FET and the whole conductive channel is uniformly p-doped with *V*_G_ ≤ 0 V (Figures 2g_1_ and 2g_4_). CdS is more sensitive to light than BP, when light illumination is introduced, a large number of electron-hole pairs will be generated in CdS flake with electrons flowing into BP and holes trapped in CdS, due to the interface barrier between BP and CdS, as well as the defects and surface state in CdS. However, the photogenerated carriers in BP should be negligible due to weak light response (inset of Fig. 2c). The trapped holes will further produce mirror-imaged charges in BP, thus leading to the up shift of the Fermi level in BP. Since BP is naturally p-doped, the up shift of the Fermi level will lead to the decrease of hole doping concentration and even the transition of the doping type into n-type, resulting in the negative response towards light irradiation (Figures 2g_2_ and 2g_5_). In addition to defect trapping, the absorbed molecules in the nanostructure surface may also be one of the reasons for negative photoresponse. During the device fabrication process, BP and CdS will inevitably contact with air, PMMA, acetone and other solutions, which may cause molecular adsorption on the surface of nanostructures, resulting in the occurrence of negative photoresponse phenomenon. Furthermore, it should be pointed out that since both BP and CdS exhibit positive photoresponse, the negative photoresponse should be reasonably originated from the heterojunction caused by defect trapping or molecular adsorption. We also notice that the device is very sensitive to light. As depicted in Fig. 2c, a weak light of 0.16 μW cm^−2^ will lead to a photocurrent (ΔI) variation of about 20 μA and thus the photoresponsivity (R) can be deduced a high value.

Figure S4 shows more designed devices, where the on-state current varies from 30 to 90 μA from device to device. It can be reasonably attributed to the difference in channel size and the thickness of BP. Shorter channel length, larger channel width and thicker thickness indicate larger on state current. After normalization of the channel width and length, we can thus deduce that the current (I*L/W) increases with the increasing of the channel thickness (Fig. S4c and Table S1). In order to eliminate the influence of these factors on the device performances, we constructed the device array with similar channel thickness, width and length. As shown in Fig. S5 and Fig. S6, both the dark current and photoresponse behavior are similar with the same BP flake and similar channel size, indicating nice repeatability and consistency of device array. In further studies, large-scale device array may be achieved through combing BP arrays and CdS arrays by developing compatible CVD preparation methods or large-scale transfer technologies. Fig. S7 shows that the photocurrent (*I*_ph_) and photoresponsivity (R) of the device, where *R*_max_ are all in the range of 10^8^–10^9 ^A W^−1^, indicating that the devices have stable and excellent photoresponse. Furthermore, the extracted R from devices with different thickness of materials is summarized in Fig. S8, showing that there is no obvious dependence between R and the thickness of heterojunction materials. We also summarize the responsivity (*R*) corresponding to the reported photodetectors constructed of different material systems, including single material^11,12,24–26^, inorganic heterojunction^14,15,23,27–34^ and organic-inorganic heterojunction^35–37^, and the results are shown in Fig. 2e. It can be concluded that the responsivity of the BP/CdS HJ-FET is among the highest in the reported devices so far. When light illumination is removed, the trapped holes can be well maintained in CdS (Fig. 2g_3_, g_6_), leading to small current state be well kept in the channel (Fig. 2f). Such charge storage behavior is similar to the long-term plasticity (LTPL) in biological synapse and is prerequisite to ensure reliable study on the synaptic photoresponse. Meanwhile, the observed current plasticity in BP/CdS stimulated by light further enables the photonic synaptic device application.

### Typical synaptic behavior of artificial photonic synapse

**Fig. 3:**
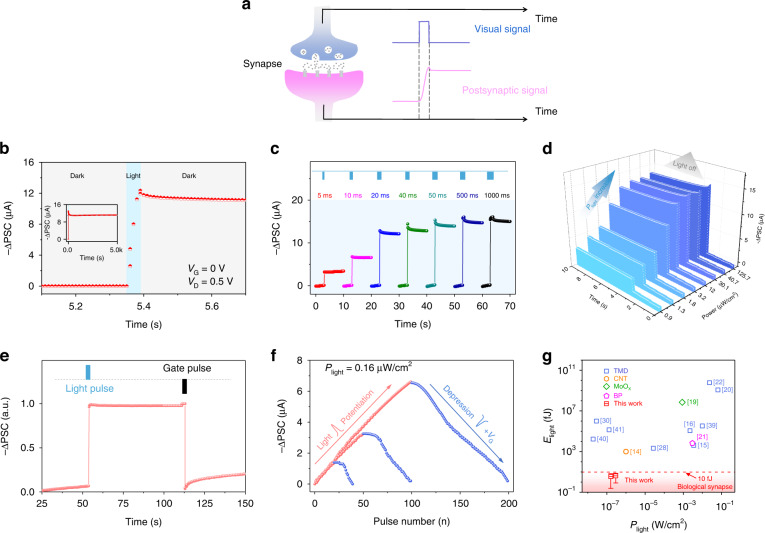
Typical synaptic behavior of artificial synapse. **a** Schematic diagram of optical signal for photonic artificial synapse. **b** Photo-activated postsynaptic current (−ΔPSC) of the artificial synapse at *P*_light_ = 11.97 μW cm^−2^ and *V*_G_ = 0 V with light pulse width at 50 ms. Inset: −ΔPSC with longer monitoring time, it remains well after 5000 s. **c** −ΔPSCs under different light pulse width at a fixed *P*_light_ of 11.97 μW cm^−2^, *V*_D_ = 0.5 V, and *V*_G_ = 0 V. **d** −ΔPSCs under different *P*_light_ at a fixed light pulse width of 50 ms (*V*_D_ = 0.5 V, *V*_G_ = 0 V). **e** The programming and erasing process of persistent PSC by light pulse and positive *V*_G_ pulse. **f** LTP/LTD characteristics trained by 20/50/100 consecutive potentiation pulses and 20/50/100 depression pulses. Potentiation process: light pulses; illumination time, 5 ms; light power, 0.16 μW cm^−2^. Depression process: electrical spikes; pulse time, 5 ms; voltage amplitude, uniformly increasing gate voltage from 0 to 4 V. **g** The summary of energy consumption in single photonic programming process (*E*_light_) of artificial photonic synapses with different architecture

The signal transmission between biological neurons is governed by the exocytosis of neurotransmitters from the presynaptic membrane to the receptor on the postsynaptic membrane. The constructed artificial synaptic device can effectively imitate this behavior with light irradiation and the corresponding schematic diagram is shown in Fig. 3a. The channel conductivity (synaptic weight) of device can be effectively modulated by light illumination, and well maintained after removing light source (Fig. S9), which is the essential feature of multimodal plasticity in photonic artificial synapse. When the device is stimulated by 450 nm light pulse (*P*_light_ = 11.97 μW cm^−2^; pulse width: 50 ms; gate voltage: 0 V), the photo-activated postsynaptic current (PSC) shows a marked negative increment (defined as −ΔPSC) by 12.5 μA and well maintained at current level of 11 μA for a long time after turning off the light source (Fig. 3b and inset of Fig. 3b), indicating nice long-term potentiation (LTP) behavior. Photoresponse behavior of the synaptic device monitored under light illumination with different wavelength is shown in Fig. S10, the artificial synapse can recognize optical signals with wavelength of 450 nm, but has no obvious response to the light at wavelengths of 633 nm and 980 nm. This can be reasonably understood that CdS has a bandgap of 2.4 eV, which can only give response to incident light with wavelength lower than 515 nm. We also monitored the LTP behavior of the synapse at 450 nm light pulse with different light stimulation information, including illumination time and intensity. As shown in Fig. 3c, the peak current value of −ΔPSC increases gradually from 3 to 16 μA with the illumination time increasing from 5 to 1000 ms (*V*_D_ = 0.5 V, *P*_light_ = 11.97 μW cm^−2^). That is to say, longer light exposure time will effectively enhance the stimulus effect, leading to larger −ΔPSC. Similarly, larger illumination power can also enhance the stimulus effect. As shown in Fig. 3d, maximum −ΔPSC increases from 2.5 to 14.5 μA with *P*_light_ increases from 0.9 to 126 μW cm^−2^ (*V*_D_ = 0.5 V, illumination time fixed at 50 ms). The peak current of −ΔPSC versus pulse width and illumination power is extracted in Fig. S11 and the results indicate that both light illumination time and intensity can effectively modulate the synapse behavior. We also monitored the synaptic behaviors by increasing the stimulated light pulse numbers. The amplitude of −ΔPSC gradually increases from 7.3 to 9.8 μA as changing the light pulse number from 1 to 50 (Fig. S12), which reflects long-term synaptic weight change of the artificial photonic synapse and the characteristics of −ΔPSC can also be effectively modulated by different light pulse number.

Moreover, the FET-based device architecture also enables the synergistic modulation of the synaptic behaviors with the assistant of negative gate voltage. As shown in Fig. S13, with larger negative gate voltage, larger maximum −ΔPSC values will be obtained, which can be reasonably attributed to higher charge separation efficiency driven by the vertical electrical field. Fig. S14 presents the detailed synaptic behavior of the device with negative *V*_G_ (−40 V), where similar larger −ΔPSC is obtained as compared with Figs. 3c and 3d. The characteristics of −ΔPSC under consecutive light pulses at different *V*_G_ are shown in Fig. S15, the −ΔPSC increases linearly with increasing the pulse number at first and then tends to saturate at a definite value with a fixed gate voltage. For example, as shown in Fig. S15a, the amplitude value of −ΔPSC linearly increases to 14 μA after five pulses, and stabilizes at 16 μA after tens of pulses (*P*_light_ = 5.32 μW cm^−2^; pulse width, 50 ms; *V*_G_ = 0 V). The amplitude rate (defined as *A*_n_/*A*_1_, where *A* is the amplitude of the −ΔPSC peak value) reaches 356% after 100 consecutive light pulse stimulations. It also indicates that the saturation current of −ΔPSC increases corresponding with similar light stimulation process by applying larger negative gate voltage (Fig. S15b, S15c). Another important parameter of the conductance margins (*G*_max_/*G*_min_, defined as the ratio between the maximum and minimum conductance value, which is one of key parameters to determine the accuracy of image recognition) are also extracted and shown in Fig. S15d. The *G*_max_/*G*_min_ is extracted to be 132 at *V*_G_ = 0 V, and can be further promoted to 804 with gate voltage of −40 V, enabling more efficient training and pattern recognition in a neuromorphic systems^38^. The synaptic device trained by lower light intensity and shorter pulse time is shown in Fig. S16 (*P*_light_ = 0.29 μW cm^−2^; pulse width, 10 ms; *V*_G_ = 0 V), presenting similar synaptic behavior but larger amplitude rate (2500%) and good linear relationship.

On the contrary, a positive gate voltage in dark condition will drive electrons into CdS to recombine with trapped holes and thus lead to the decrease of −ΔPSC, indicating depression process of the synapse (Fig. 3e). Thus, On the basis of the synergistic effect of optical programming and electrical erasing in the artificial synapse, optical-stimulation-induced long-term potentiation (LTP) and electrical-response-driven long-term depression (LTD) are successfully simulated. Firstly, LTP/LTD characteristics are trained by 50 consecutive potentiation pulses (optical programming) and depression pulses (electrical erasing) under different light intensity on SiO_2_/Si substrate (Fig. S17), the curves show good linear relationship with lower light illumination (*P*_light_ = 0.29 μW cm^−2^). In order to evaluate the endurance characteristics of the device, pulse-switching characteristics and multiple cycles of LTP/LTD characteristics are tested in Fig. S18. The result exhibits that the device can be switched well between the program and erase state with more than 150 cycles over 3000 s. Moreover, the potentiation and depression processes can be continuously simulated by applying consecutive light and *V*_G_ spikes, reflecting repeatable switching and good endurance performance of the device. We also probed the synaptic behavior of BP/CdS heterostructure on h-BN/graphene substrate and the results are shown in Fig. S19, where similar synaptic properties are observed, further confirming that the synaptic behavior originates from the heterostructure rather than substrate. Figure 3f depicts the measured LTP/LTD curves with BP/CdS heterostructure on h-BN/graphene substrate, where the LTP is triggered by 20/50/100 consecutive light pulses (illumination time, 5 ms; light power, 0.16 μW cm^−2^) and the LTD is elicited by same number of electrical spikes (pulse time, 5 ms; voltage amplitude, uniformly increasing gate voltage from 0 to 4 V). The result shows symmetric and linear LTP and LTD process, demonstrating great optical and electrical controllability of the synaptic device, which is also the basis for realizing high image recognition accuracy. The energy consumption for the photonic programming process and electric erasing process are further estimated to be about 8.9 fJ and 25 fJ per spike (detailed calculation and extraction process of the energy consumption is shown in Note S1), respectively, which is comparable to the biological synapse (10 fJ) and compares favorably to most of the reported synaptic devices (e.g., TMD^15,16,20,22,28,30,39–41^, CNT^14^, MoO_X_^19^ and BP^21^ based synaptic device), indicating potential application in energy efficient neuromorphic systems (Fig. 3g). The artificial photonic synapse clearly exhibits photonic potentiation and electronic depression behaviors, indicating that the artificial synaptic devices support optical-write and electronic-erase functions for learning and recognition in artificial neural networks.

### Simulation of FONN for image recognition

**Fig. 4:**
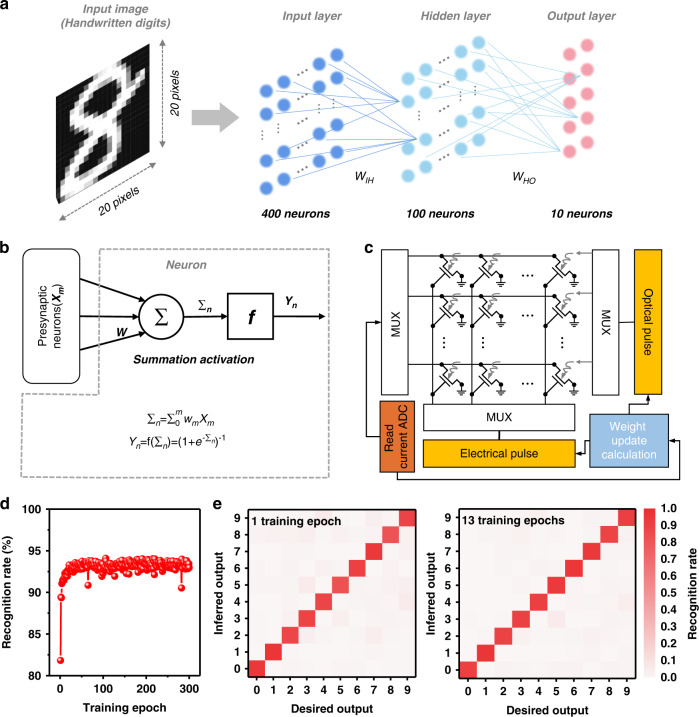
Simulation of FONN for image recognition by the artificial photonic synapse. **a** Schematic illustration of the simulated FONN with a three-layer architecture. **b** Schematic of a neuron node and synaptic weight update process. **c** Circuit block diagram for the simulation showing the photonic synapse array and the peripheral circuits. MUX, multiplexer; ADC, analog-to-digital converter. **d** Calculated MNIST recognition rate as a function of training epochs. **e** The confusion matrix between desired value and predicted value after 1 and 13 training epochs

To evaluate the learning capability of our low-power photonic synaptic devices, a fully-connected optoelectronic neural network (FONN) is constructed with a three-layer architecture for MNIST handwritten digit recognition. As demonstrated in Fig. 4a, the network consists of an input layer (400 neurons, corresponding to 20 × 20 pixels of an input image), a hidden layer (100 neurons) and an output layer (10 neurons, corresponding to the 10 classes of recognized digits 0~9). Here, each neuron in the network receives the weighted summed results through the summation function (Σn) from the previous layer and pass the output value by an activation function (Yn), which is as shown in Fig. 4b. The circuit block diagram for the simulation is illustrated in Fig. 4c, including the simulated photonic synapse array and the peripheral circuits. The weight update calculation is based on inner product of the input signal vector and synapse matrix that are read by the read current ADC module, providing the feedback into the simulated synapse array to update the synaptic weight via optical or electric pulses (see experimental methods for more details). After training the FONN with 6000 handwritten images, the recognition rate test is carried out with a separate testing set (with 1000 images), and the results is shown in Fig. 4d. The recognition rate of our FONN simulation can achieve 93.2% on average (94.1% as a maximum). It is worth noting that the recognition rate rises quickly during the initial three training epochs and the recognition rate could be up to 81.8% at the first epoch, which is significantly outperformed the previous results using the same datasets^15,21,39,42–44^. The confusion matrix for the recognition rate test is present in Fig. 4e, indicating that the FONN can highly-accurately recognize every classes of digits (0~9) since the initial epoch.

## Discussion

In summary, we successfully fabricated an artificial photonic synapse based on BP/CdS heterojunction with multilayer BP as conducting channel and CdS flake as the light-sensitive layer. Due to the effectively charge transfer between BP and CdS, typical photonic synaptic behaviors can be effectively modulated under the synergistic effect of light pulses and electrical pulses, including photosensitivity, postsynaptic photocurrents and persistent photoconductivity. Based on the synaptic characteristic of LTP/LTD curve under photonic programming and electric erasing process, a FONN is constructed for image recognition against the Modified National Institute of Standards and Technology (MNIST) handwriting image dataset, with recognition accuracy up to 94.1% and energy consumption as low as 0.43–8.9 fJ per light spike and 25 fJ per electrical spike. The proposed artificial photonic synapse provides a promising concept that use 2D heterojunctions for neuromorphic computation, machine vision and artificial intelligence systems.

## Materials and Methods

### Device fabrication

The heterojunctions were fabricated by a dry transfer technique^45,46^. The CdS flakes were synthesized by a vapor growth strategy reported previously^23,33^, and then were transferred onto the silicon substrate with 300-nm silicon oxide by polydimethylsiloxane (PDMS) stamp using a three-dimensional transfer platform. Next, multilayer BP flakes were mechanically exfoliated onto a PDMS film and transfer on top of the CdS flake after aligning under an optical microscope. Finally, standard e-beam lithography (EBL, Raith 150 Two) was employed to define the source and drain patterns, Au/Cr (50 nm/10 nm) metal contacts were then deposited by using metal thermal evaporation with a standard lift-off process.

### Material and performance characterization

The optical images of simples were obtained by using a polarizing microscope (ZEISS, Axio Scope A1). The SEM study was characterized by a ZEISS Sigma HD instrument. The morphology of the devices was confirmed by an atomic force microscopy (AFM, Bruker Dimension Icon) in a tapping mode. Photoluminescence and Raman measurements were performed by using a confocal μ-PL system (WITec, alpha-300) with a 488 nm laser excitation source. All the electrical properties of the artificial photonic synapses were characterized in high vacuum (10^−4^ Pa) with an Agilent-B1500 semiconductor parameter analyzer and a Lakeshore probe station at room temperature. The light illumination was applied by a 450 nm laser and controlled by a laser controller (Thorlabs, ITC4001). The optical power was measured with Thorlabs’ Optical Power Meter.

### Simulation of FONN for handwritten digit recognition

The simulation is carried out based on the “NeuroSim+” simulation platform, which could provide system-level simulation including device level (transistor technology and memory models) to the circuit level (synaptic array architecture and peripheral neuron circuits)^47,48^. The analog conductance change of the synaptic device is fitted by an exponential model to mimic the weight update behavior in the neural network using the following equations:

$$G_{{\rm{LTP}}} = G_{{\rm{min}}} + \frac{{(G_{{\rm{max}}} - G_{{\rm{min}}})(1 - {\rm{e}}^{ - \frac{P}{{{\rm{NL}}}}})}}{{1 - {\rm{e}}^{ - \frac{{P_{{\rm{max}}}}}{{{\rm{NL}}}}}}}$$

$$G_{{\rm{LTD}}} = G_{{\rm{max}}} - \frac{{(G_{{\rm{max}}} - G_{{\rm{min}}})(1 - {\rm{e}}^{\frac{{P - P_{{\rm{max}}}}}{{{\rm{NL}}}}})}}{{1 - {\rm{e}}^{ - \frac{{P_{{\rm{max}}}}}{{{\rm{NL}}}}}}}$$

where variations of *G*_LTP_, *G*_LTD_, and P are the conductance for long-term potentiation (LTP), long-term depression (LTD) and pulse number, respectively. And the constant of *G*_max_, *G*_min_, *P*_max_, NL represent the maximum conductance, minimum conductance, maximum pulse number between the maximum/minimum conductance state, and a non-linear parameter of the weight updating, respectively. By using the equations above, NL are firstly extracted from the data in Fig. 3f. After loading the 6000 handwritten images from MNIST datasets, the training algorithm including the feed forward (FF) and back propagation (BP) is carried out. Subsequently, the testing set consisting of 1000 images are loaded to test the recognition accuracy of the network. Here, the input images from MNIST database are all binarized with a threshold of 128 for every pixel and cropped with the size of 20 × 20 pixels. The activation function in the neurons is the sigmoid function.

## Supplementary information

Supplementary Information for Optical synaptic devices with ultra-low power consumption for neuromorphic computing

## Data Availability

The data that support the findings of this study are available from the corresponding authors on reasonable request. Source data are provided with this paper.
