# Supplementary material for: Optical synaptic devices with ultra-low power consumption for neuromorphic computing
Source: Light Sci Appl. 2022 Nov 29;11:337. doi: 10.1038/s41377-022-01031-z (PMC9705294; doi:10.1038/s41377-022-01031-z)
Supplement: Supplementary file 1 — Supplementary Information for Optical synaptic devices with ultra-low power consumption for neuromorphic computing [file 41377_2022_1031_MOESM1_ESM.docx]

Supplementary Information for

**Optical synaptic devices with ultra-low power consumption for neuromorphic computing**

Chenguang Zhu^1,2,†^, Huawei Liu^1,2,†^, Wenqiang Wang^1,2,†^, Li Xiang^1,2,*^, Jie Jiang^3^, Qin Shuai^1,2^, Xin Yang^1,2^, Tian Zhang^1,2^, Biyuan Zheng^1,2^, Hui Wang^1,2^, Dong Li^1,2,*^ and Anlian Pan^1,2,*^

^1^Key Laboratory for Micro-Nano Physics and Technology of Hunan Province, State Key Laboratory of Chemo/Biosensing and Chemometrics, College of Materials Science and Engineering, Hunan University, Changsha 410082, China

^2^Hunan Institute of Optoelectronic Integration, Hunan University, Changsha, 410082, China

^3^School of Physics and Electronics, Central South University, Changsha, 410083, China

^†^These authors contributed equally to this work.

*E-mail: xiangli93@hnu.edu.cn; liidong@hnu.edu.cn; anlian.pan@hnu.edu.cn.

1. **Materials characterization of BP, CdS, and BP/CdS heterostructure**

The optical image is shown in Fig. S1a, CVD CdS is firstly transferred onto the SiO_2_/Si substrate by typical polydimethylsiloxane (PDMS)-assisted dry transfer method. Few-layer BP is mechanically exfoliated and transferred onto the CdS flakes with same method. The AFM image is shown in Fig. S1b, the result indicates that the thicknesses of BP and CdS are ~16 nm and ~33 nm, respectively. The Raman spectra of BP and PL spectra of CdS are excited by 488 nm laser. Fig. S1c shows that three typical Raman peaks of BP are located at 365 cm^-1^ (A_g_^1^ peak), 440 cm^-1^ (B_2g_ peak), and 470 cm^-1^ (A_g_^2^ peak), respectively. Fig. S1d exhibits typical PL emission characteristic of CdS with emission peak at 515 nm. The results are consistent with previously reported work^1-5^.


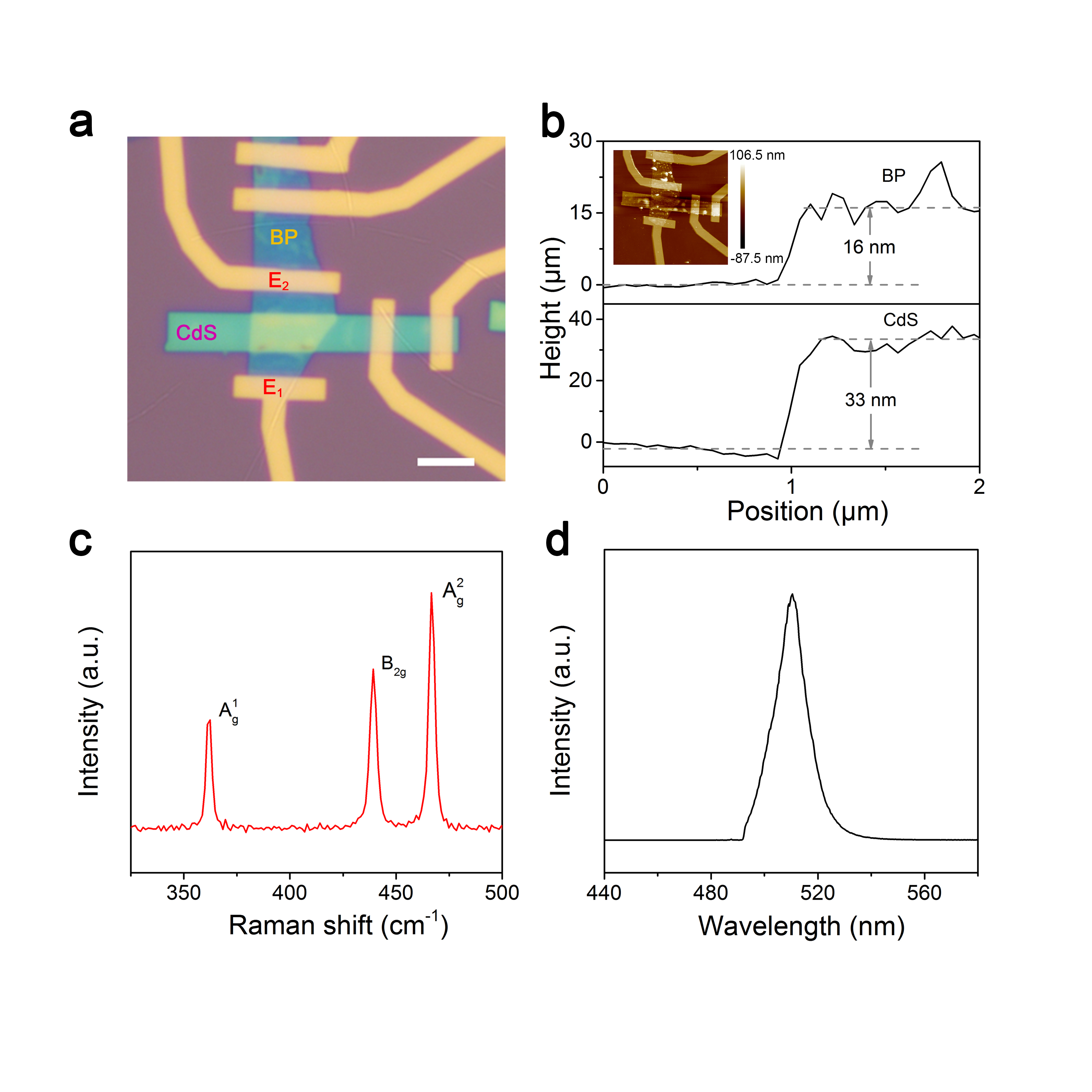


**Figure S1. Material characterization of artificial photonic synapse.** **a** Optical image of the device; scale bar, 5 μm. **b** Atomic force microscopy (AFM) image and height information of BP and CdS. **c** Raman spectrum of BP. **d** PL spectrum of the CdS under 488 nm laser excitation.

1. **Electrical comparison of HJ-FET and BP-FET under dark condition**

It should be noted here that, for HJ-FET, the electrodes are only in direct contact with BP, but not with CdS. The HJ-FET possesses similar electrical performances with BP-FET under dark condition, indicating that CdS possesses negligible influence on BP-FET under dark condition. The mobility is calculated with equation

$\mu=\frac{dI_{D}}{dV_{G}}\times\frac{L}{W\varepsilon_{i}V_{D}}$ (1)

where *L* is the channel length, *W* is the channel width, *ε*_i_ (*ε*_i_ = *ε*_0_*ε*_r_/*d*, *ε*_r_ for SiO_2_ is 3.9, *ε*_0_ is the dielectric constant) is the capacitance per unit area between the channel and the back gate, *I*_D_, *V*_D_ and *V*_G_ stand for drain current, drain voltage and back gate voltage, respectively. As shown in Fig. S2, the *I*_max_ of HJ-FET and BP-FET is 82.9 μA and 95.4 μA, the on/off ratio of HJ-FET and BP-FET is 5.38 × 10^3^ and 7.01 × 10^3^, the calculated mobility of HJ-FET and BP-FET is 352 cm^2^ V^-1^ s^-1^ and 322 cm^2^ V^-1^ s^-1^.


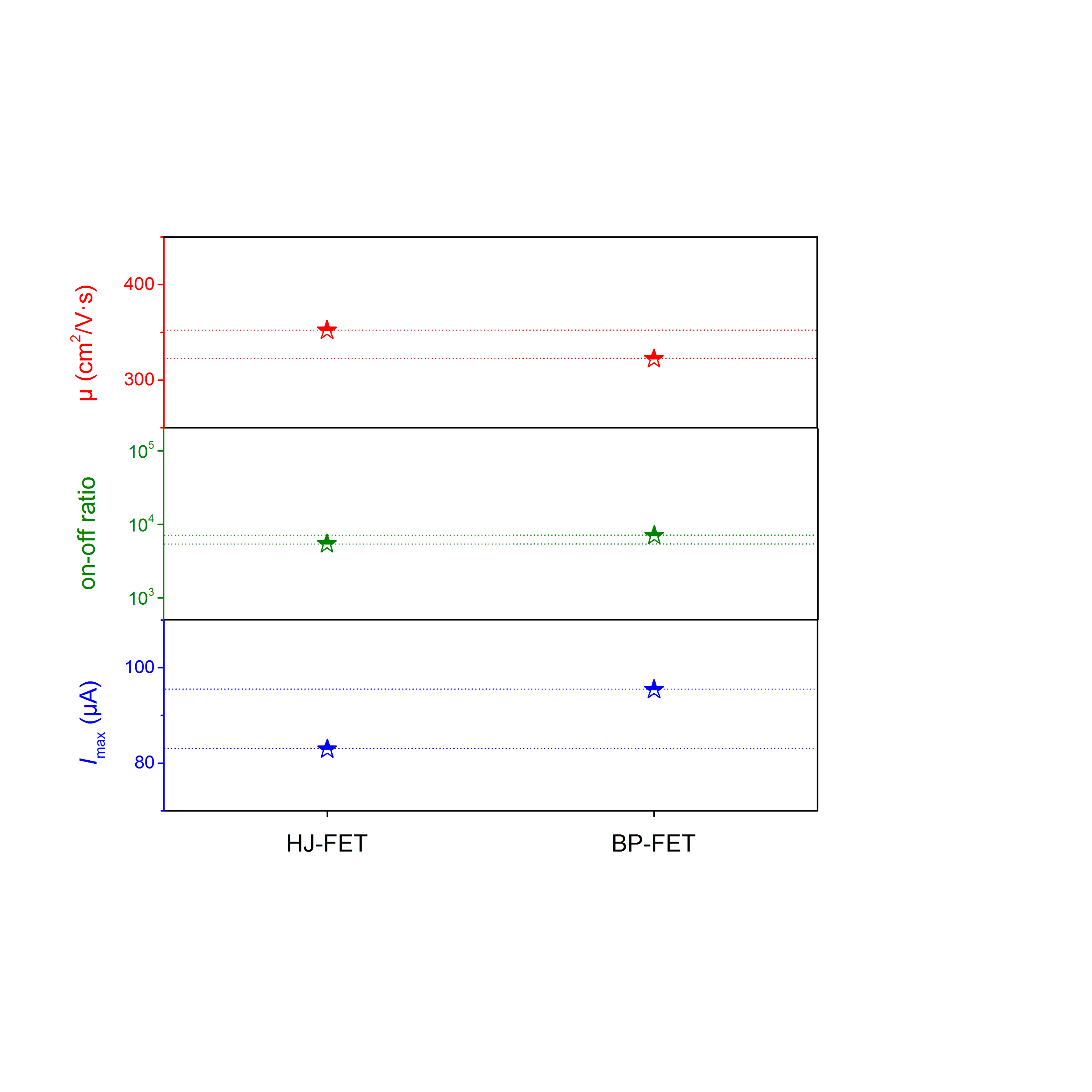


**Figure S2.** Comparison of electrical properties between HJ-FET and BP-FET under dark condition.

1. **The optoelectronic characteristics of the CdS-FET**

The photoresponsivity (R) of the transistor can be evaluated from the equation of R = *I*_ph_/(*P*_light_ × A), where *I*_ph_ is the photocurrent, *P*_light_ is the light power density, and A is the effective area of the conductive channel. The results indicate that the n-type CdS shows ultra-sensitive and positive photoresponse with *R*_max_ approaching 10^8^ A W^-1^.


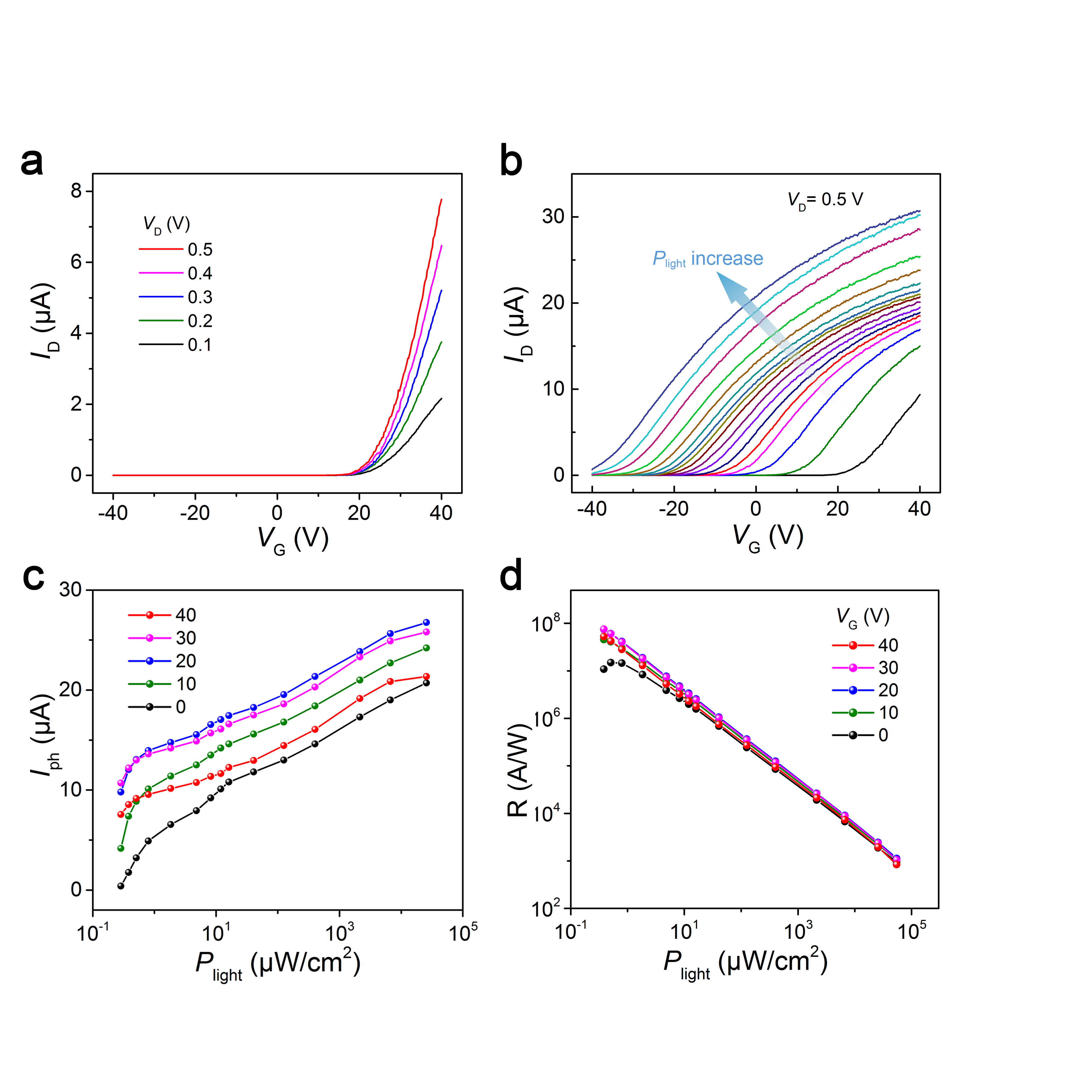


**Figure S3.** **The optical response of the CdS-FET. a** Transfer curves of CdS-FET under dark condition in linear coordinate, showing typical n-type behavior. **b** Transfer curves of CdS-FET under different light power illumination in linear coordinate, showing strong and positive light response behavior. **c** Photocurrent and **d** photoresponsivity of CdS-FET under various illumination power intensities at different *V*_G_.

1. **Repeatability test of the HJ-FETs**

Several devices are constructed and tested to verify the repeatability of the negative photoresponse characteristic of HJ-FETs under weak light illumination (0.16 μW cm^-2^). The results in Fig. S4 show that the devices exhibit different initial currents and photocurrents due to the different thicknesses and channel sizes of the heterojunction materials, Fig. S4c and Table S1 present that the largest normalized currents (I*L/W) extracted from different devices increase gradually with increasing of the thickness of BP attributed to the increased conductivity with thicker BP, but all of them have obvious negative photoresponse characteristics. As to the constructed 2×3 device array with the same thickness of material and same size of conductive channel, they show highly consistent electrical and optoelectronic performance (Fig. S5 and Fig. S6), indicating nice repeatability of the HJ-FETs.


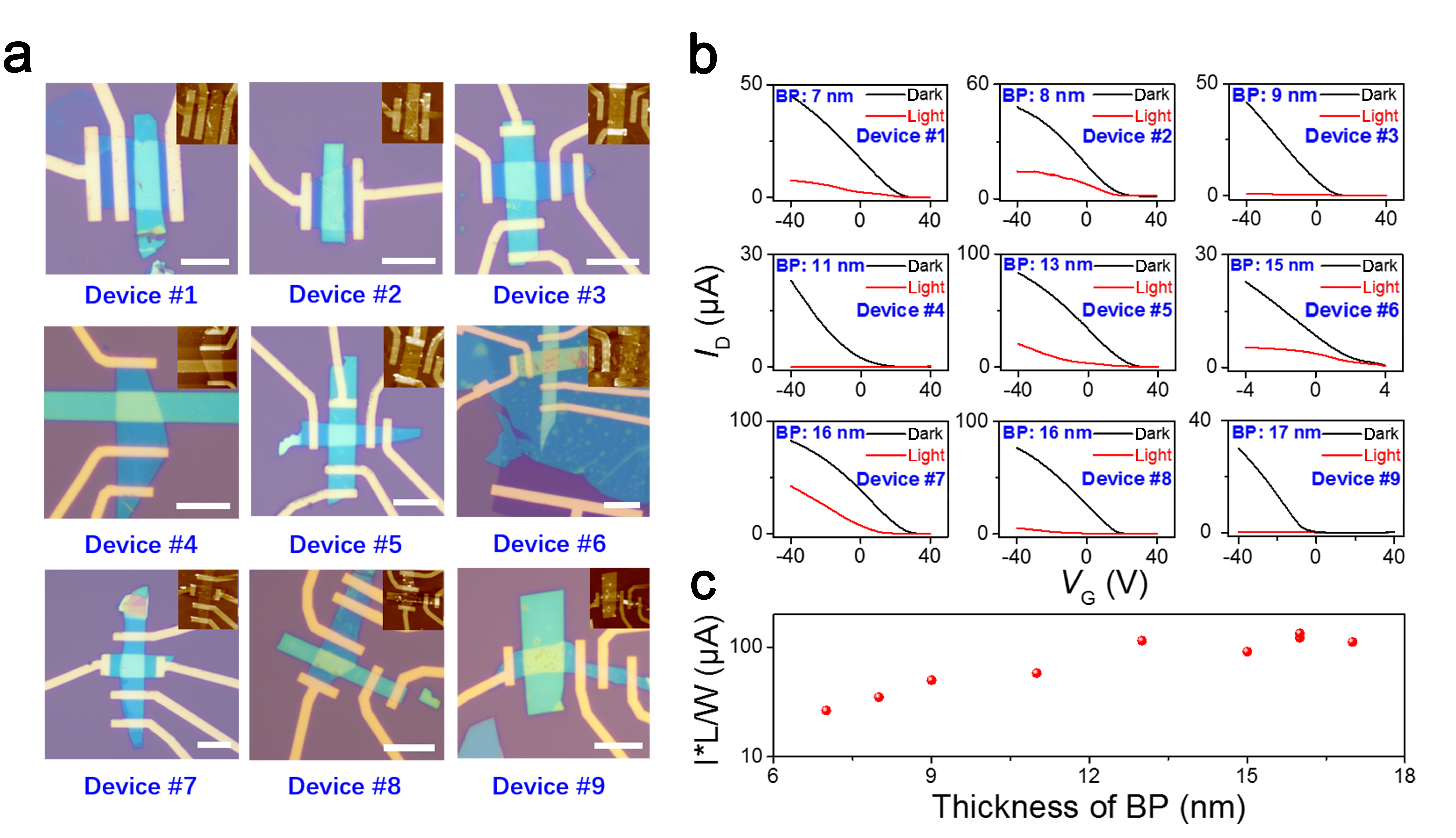


**Figure S4.** **Repeatability test of the HJ-FETs with different material thicknesses.** **a** Optical images of device #1-9. Scale bar, 10 μm. **b** The randomly selected nine HJ-FETs are repeatability tested under dark condition and weak light illumination (*P*_light_ = 0.16 μW cm^-2^). **c** The maximum current (*I*_max_) and normalized maximum current (I*L/W) of the 9 synaptic devices with different thicknesses of BP.

**Table S1. The height information and extracted *I*_max_ (I*L/W) of the nine devices in Fig. S4**

| Device number | The thickness of BP | The thickness of CdS | *I*_max_ (I*W/L) |
| --- | --- | --- | --- |
| Device #6 | 7 nm | 32 nm | 26 |
| Device #7 | 8 nm | 33 nm | 35 |
| Device #5 | 9 nm | 33.5 nm | 50 |
| Device #8 | 11 nm | 36 nm | 58 |
| Device #3 | 13 nm | 32 nm | 115 |
| Device #9 | 15 nm | 20 nm | 91 |
| Device #4 | 16 nm | 28 nm | 134 |
| Device #2 | 16 nm | 33 nm | 121 |
| Device #1 | 17 nm | 37 nm | 112 |


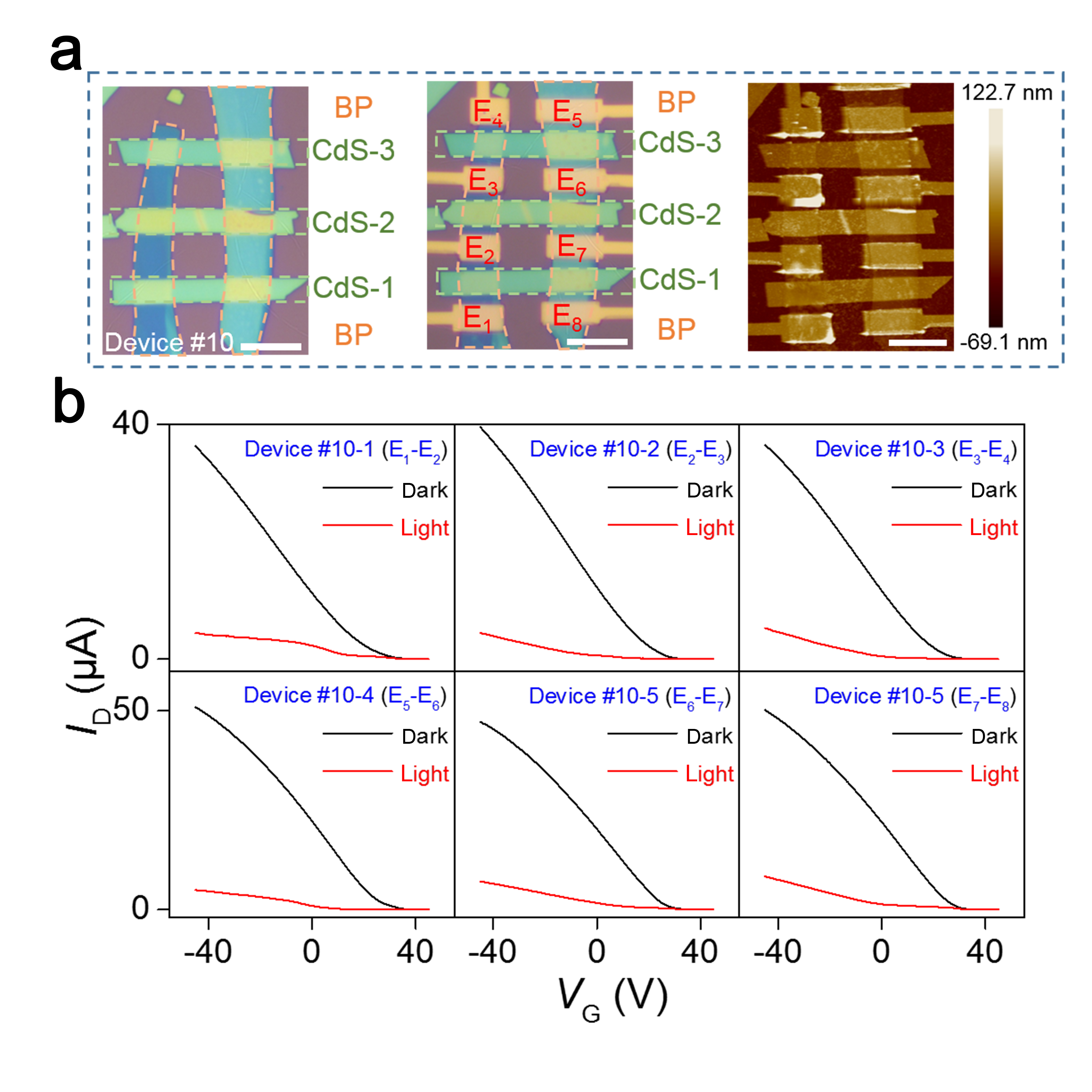


**Figure S5. Repeatability test of the 2×3 device array.** **a** Optical and AFM images of the 2×3 device array. Scale bar, 10 μm. **b** Optoelectronic properties of the 2×3 device array. Device #10-1, Device #10-2 and Device #10-3 are fabricated based on the same BP with same thickness on the left. Device #10-4, Device #10-5 and Device #10-6 are fabricated based on the same BP with same thickness on the right. All the devices have similar channel size. *V*_D_ = 0.5 V, *P*_light_ is fixed at 0.16 μW cm^-2^.


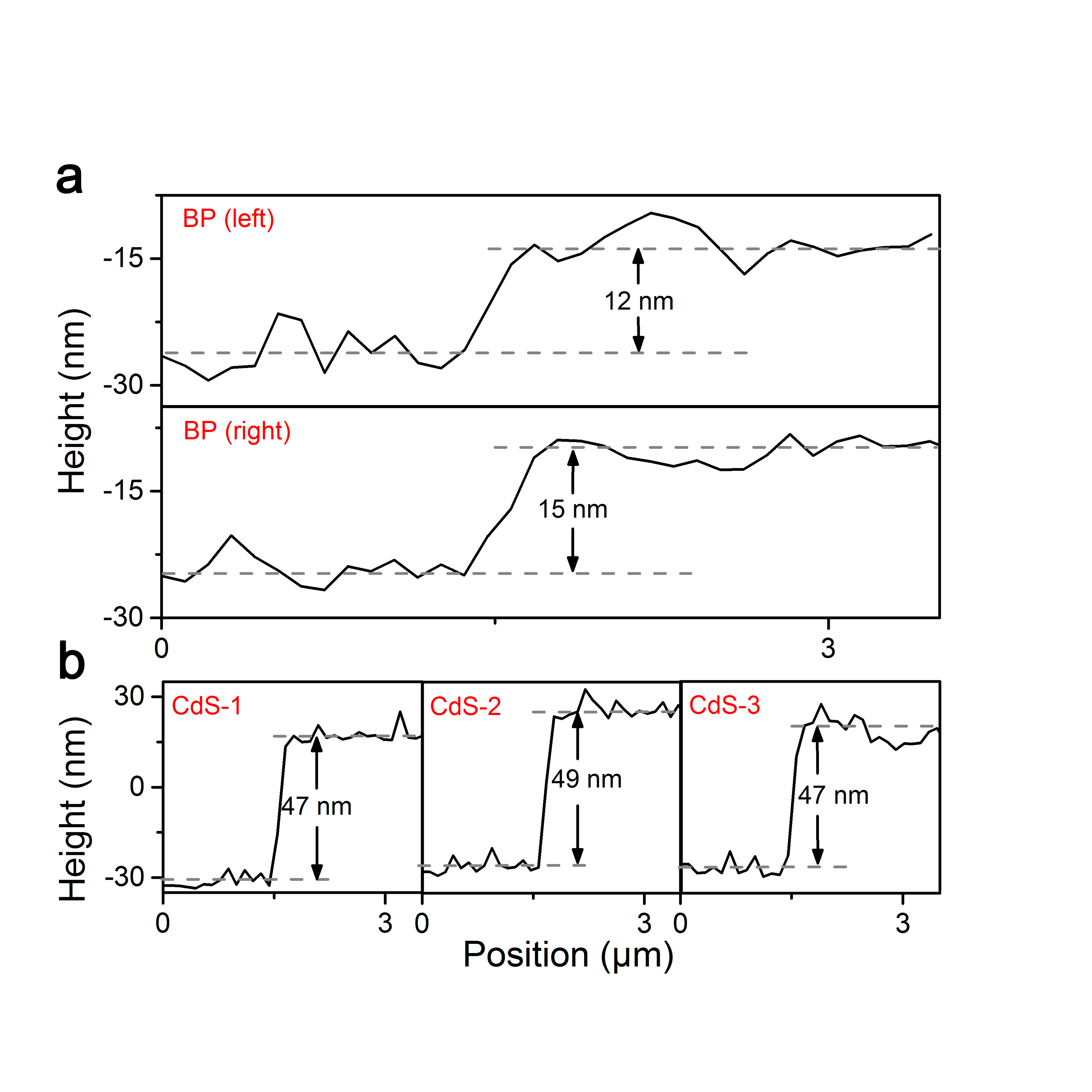


**Figure S6. Thickness information of BP and CdS in 2×3 device array** shown in Figure S5**.** **a** Height information of BP. The results show that the thicknesses of black phosphorus on the left side and right side are 12 nm and 15 nm, respectively. **b** Height information of CdS. The results show that the thicknesses of CdS-1, CdS-2 and CdS-3 are 47 nm, 49 nm and 47 nm, respectively.

1. **The extracted *I*_ph_ and R of BP/CdS heterostructure device**

The photocurrent (*I*_ph_) is defined as the current difference between the *I*_D_ in dark state and *I*_D_ under light illumination^6^. The p-type BP over CdS is n-doped when CdS traps holes under light illumination, which increases the resistance of BP and leads to the current decrement (negative *I*_ph_). The results in Fig. S7 indicate that the designed HJ-FET possesses ultra-high photoresponsivity of 10^9^ A/W. Furthermore, the extracted R from devices with different thicknesses of materials are summarized in Fig. S8, showing that there is no obvious dependence between R and the thickness of heterojunction.


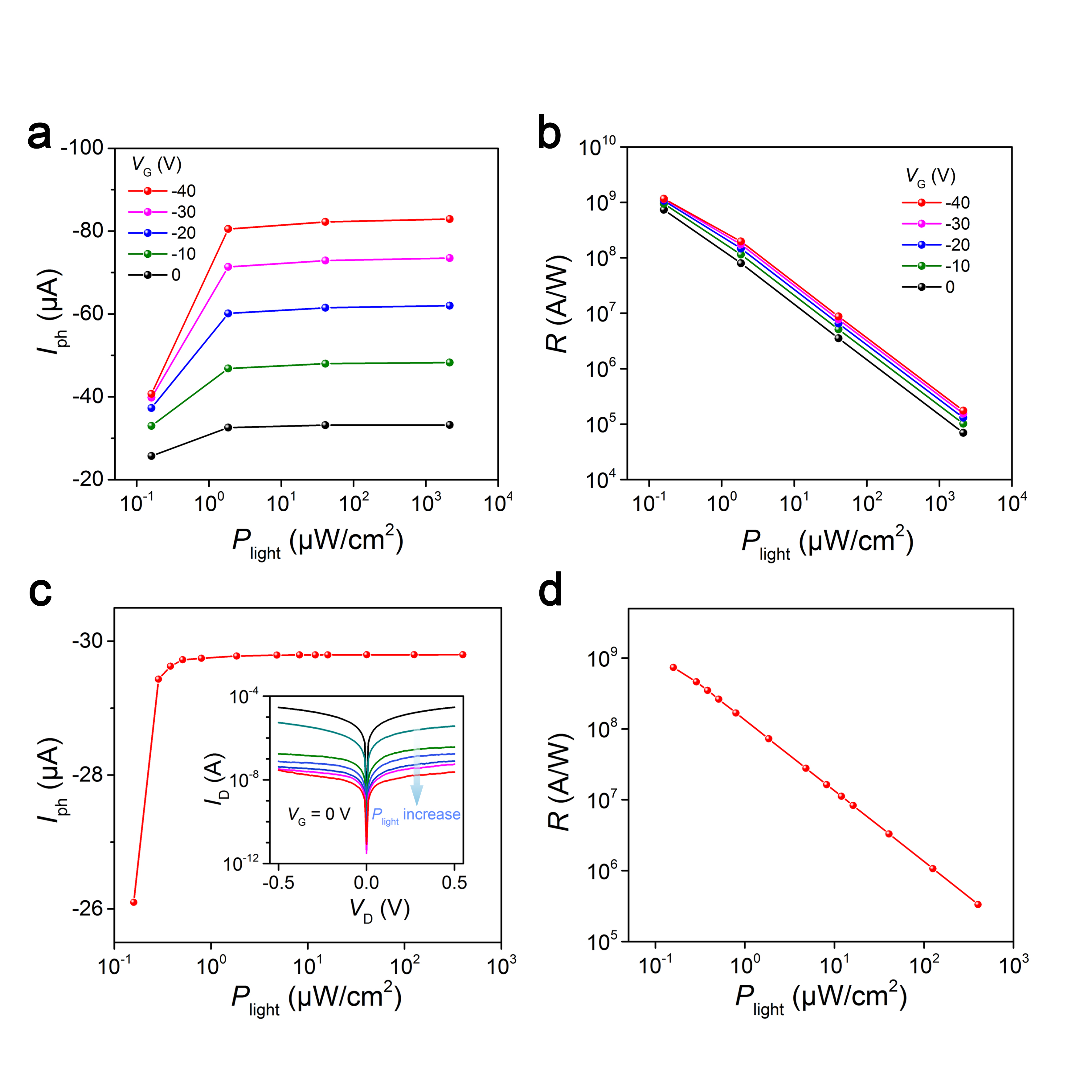


**Figure S7. The photocurrent and photoresponsivity of the artificial photonic synapse. a** Dependence of photocurrent on illumination power intensities under different *V*_G_ of HJ-FET, which is extracted from Figure 2c. **b** Photoresponsivity (R) of the HJ-FET at various illumination power intensities and different *V*_G_ according to equation R = |*I*_ph_|/(*P*_light_×A). Here, *I*_ph_ is the photocurrent, *P*_light_ is the incident light power density, and A is the effective area of the device channel, respectively. **c** Dependence of photocurrent on illumination power intensities with *V*_G_ fixed at 0 V, which is extracted from *I*_D_-*V*_D_ curves. Inset: *I*_D_-*V*_D_ curves of HJ-FET under various illumination power intensities in logarithmic coordinate with *V*_G_ fixed at 0 V. **d** The calculated photoresponsivity (R) of the HJ-FET at various illumination power intensities based on Fig. S7c.


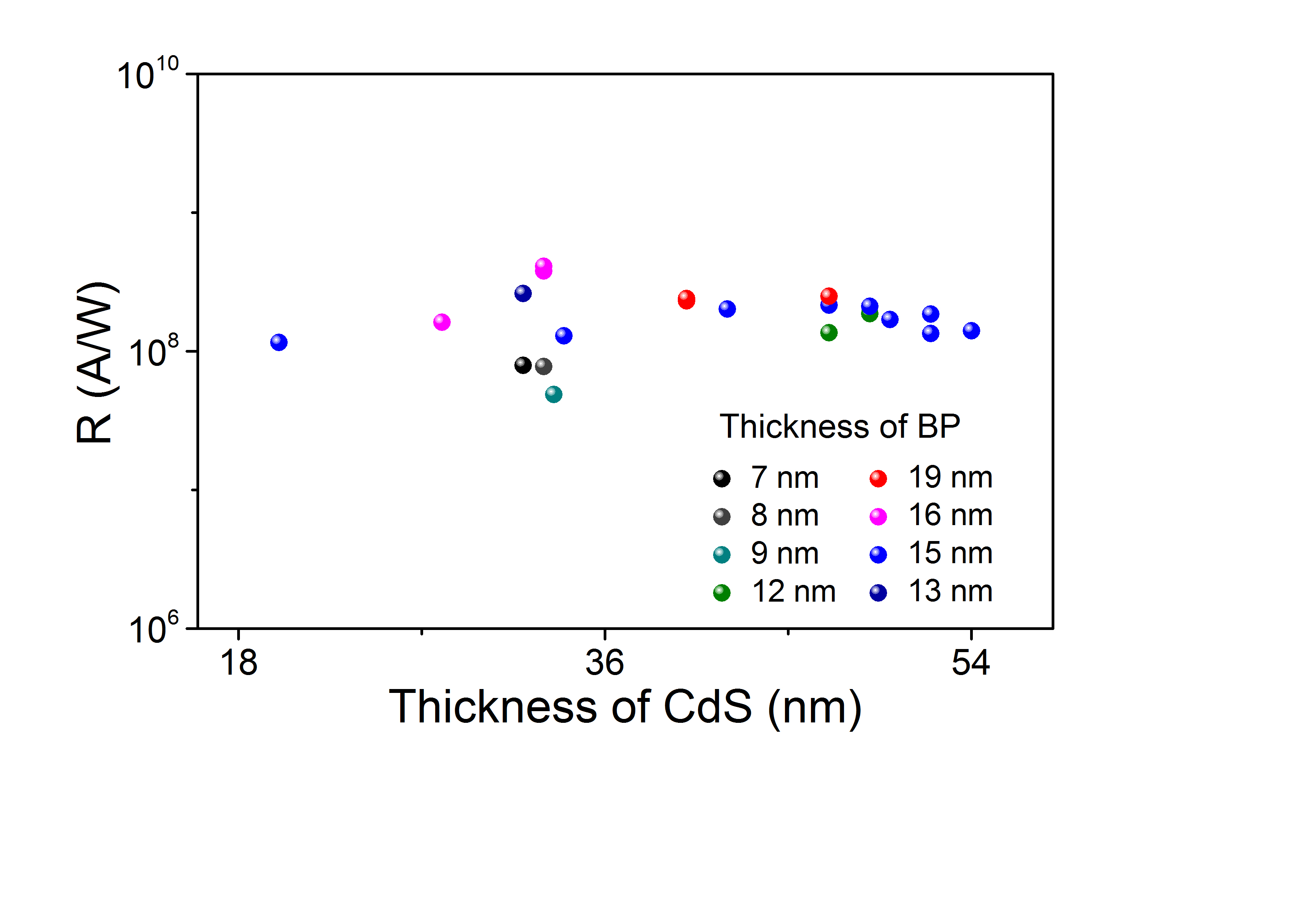


**Figure S8.** Statistical analyses of R extracted from devices with different thicknesses of materials.

1. **Retention time of the artificial photonic synapse**

To evaluate the retention time, the postsynaptic currents of artificial photonic synapse at different negative *V*_G_ under *P*_light_ = 5.32 μW cm^-2^ are measured in Fig. S9. The results show that no significant variation is observed after 1000 s under single light pulse.


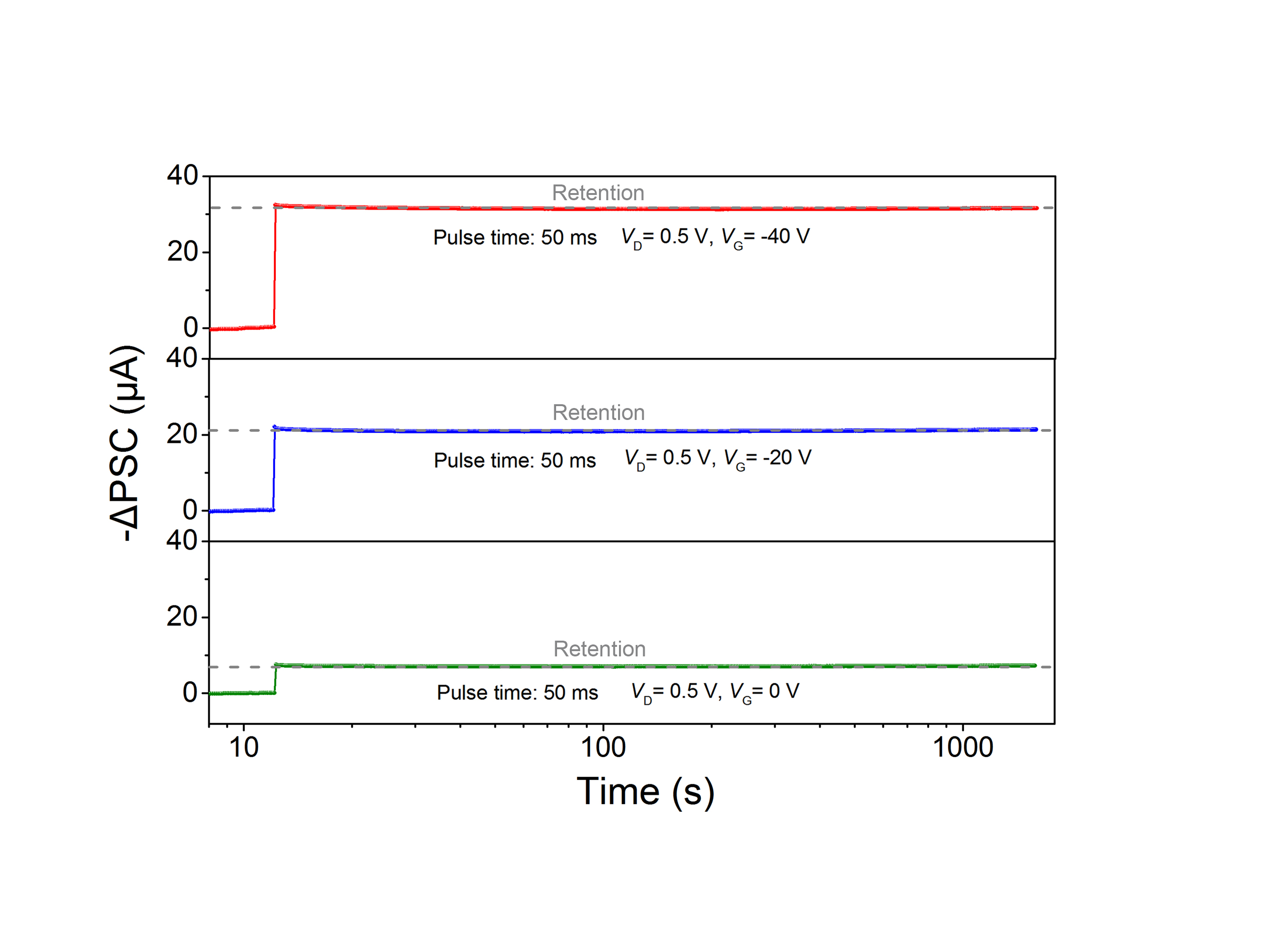


**Figure S9.** Retention behavior of the artificial photonic synapse under different negative *V*_G_.

1. **Synaptic behavior of artificial synapse with wide range wavelength**

Photoresponse behavior of the synaptic device monitored under light illumination with different wavelength is shown in Fig. S10a, the artificial synapse can recognize optical signal with wavelength of 450 nm, but has no obvious response to the light at wavelengths of 633 nm and 980 nm with *P*_light_ of 11.97 μW cm^-2^. It can be also clearly reflected by the transfer characteristics of artificial synapses shown in Fig. S10b. As can be seen, the synaptic device shows obvious negative light response under 450 nm light illumination, however, this phenomenon doesn’t appear under 633 nm and 980 nm light illumination.


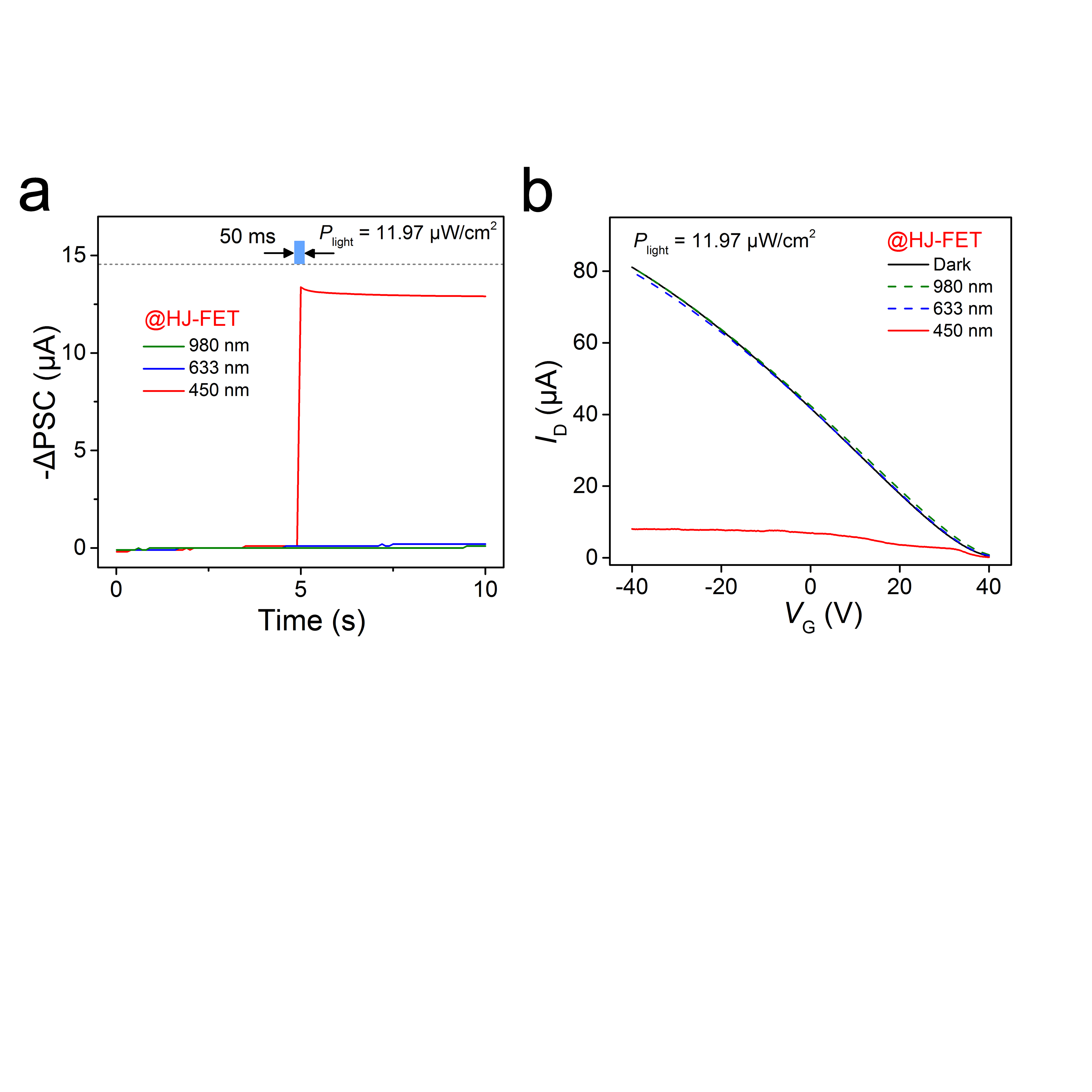


**Figure S10. Synaptic behavior of artificial synapse with wide range wavelength.** **a** Photo-activated postsynaptic current (-ΔPSC) of the artificial synapse under different wavelengths of light. *V*_D_ is fixed at 0.5 V. **b** The transfer curves of synaptic device (HJ-FET) under dark condition and different wavelength of light. The power of light is fixed at 11.97 μW cm^-2^ and *V*_D_ = 0.5 V, showing negative photoresponse at a wavelength of 450 nm.

1. **The dependence of −ΔPSCs with different light pulse width and *P*_light_**

-ΔPSC increases rapidly at first and then tends to be stable with the increase of pulse width and optical power, indicating that both light illumination time and intensity can effectively modulate the synaptic behavior.


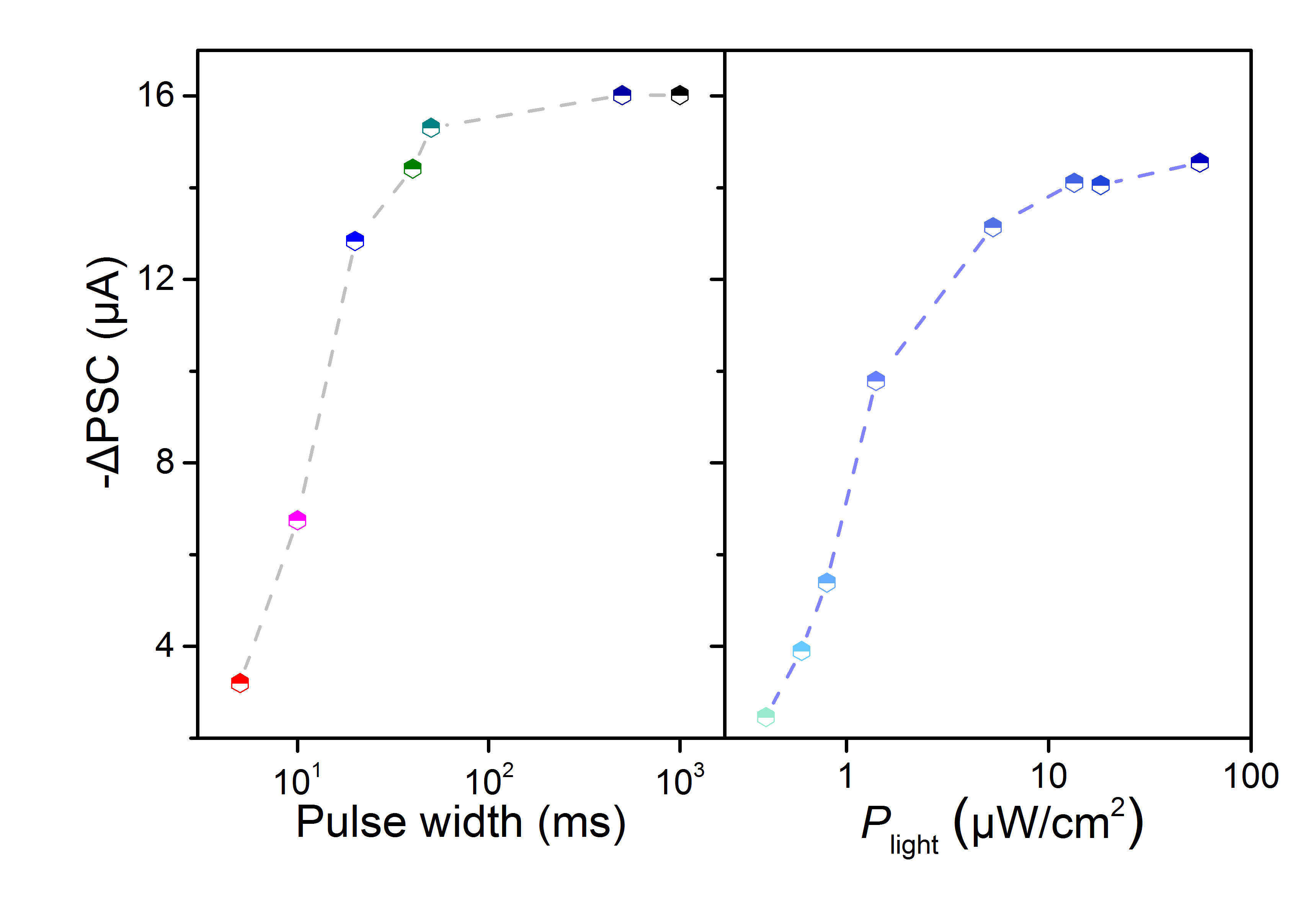


**Figure S11.** The dependence of -ΔPSCs with different light pulse width and *P*_light_.

1. **The PSC characteristics under different laser numbers**

Long-term plasticity is significantly enhanced with the increase of pulse number, corresponding to the increase of excitability index and the change of long-term synaptic weight^7^.


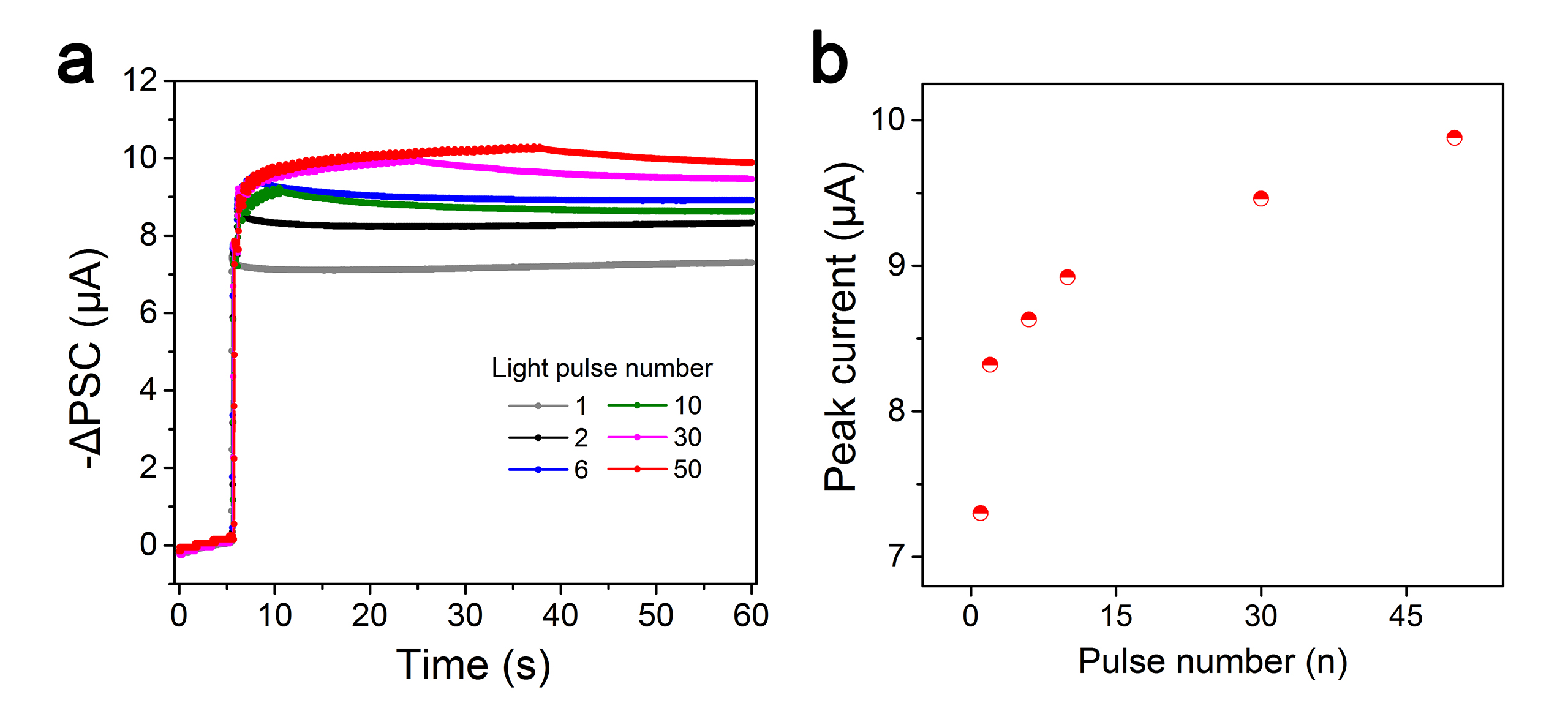


**Figure S12. The PSC characteristics under different laser numbers in optical modulation mode.** **a** The evolution of -ΔPSC under different light pulse numbers with *V*_G_ is 0 V, *P*_light_ is fixed at 5.32 μW cm^-2^. **b** The peak current under 1, 2, 6, 10, 30, 50 light pulses at *V*_G_ = 0 V.

1. **The optical response assisted with *V*_G_**

Attributed to higher charge separation efficiency driven by the vertical electrical field, larger -ΔPSC will be obtained with larger negative gate voltage applied on Si substrate.


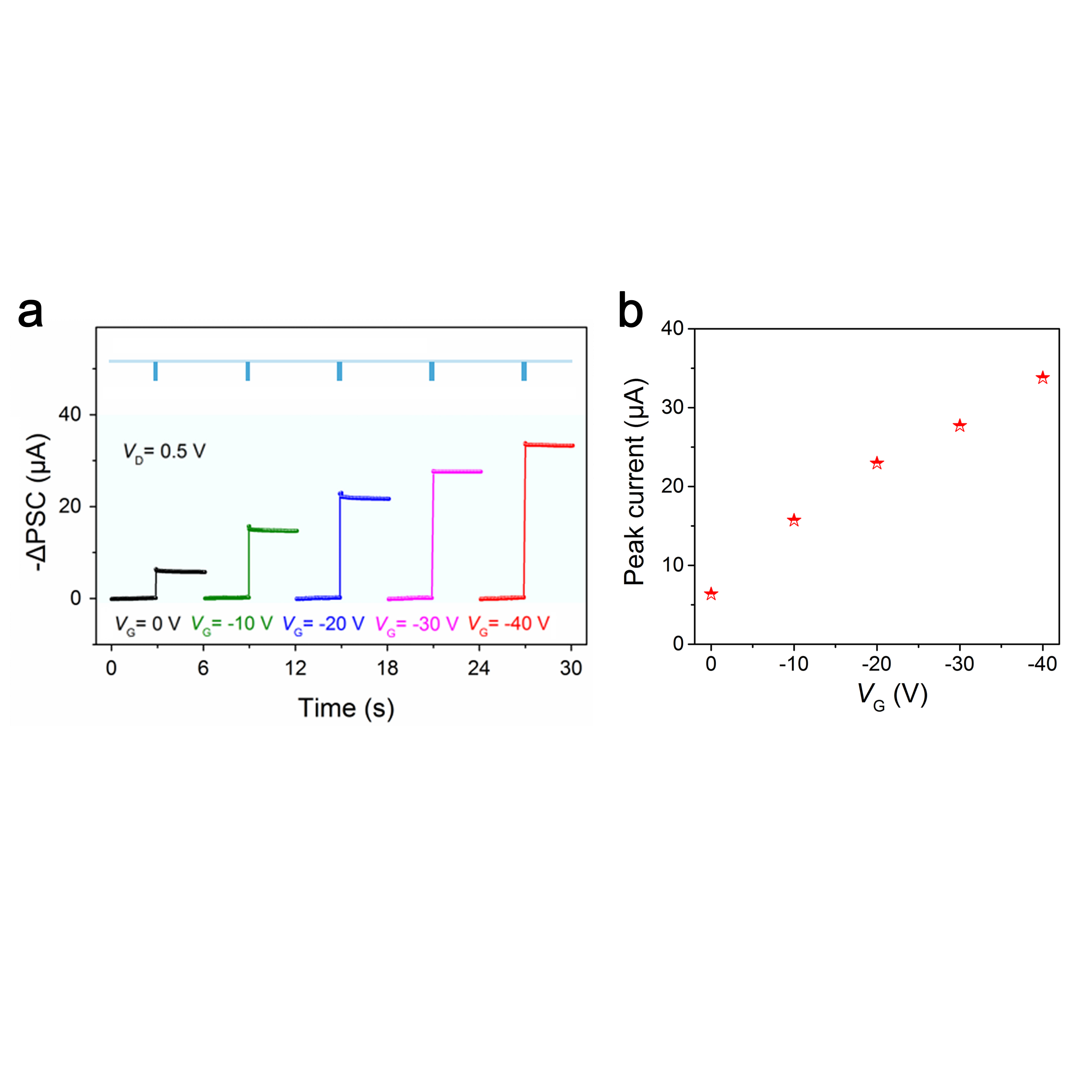


**Figure S13.** **The optical response of the artificial photonic synapse assisted with *V*_G_. a** -ΔPSC under the synergetic effect of light illumination and different *V*_G_ (0 V to -40 V) at *V*_D_ = 0.5 V. Top set: The schematic diagram of visual presynaptic signals. The light pulse width is fixed at 50 ms with *P*_light_ = 5.32 μW cm^-2^. **b** The peak current of -ΔPSC as a function of *V*_G_.

1. **Synergistic effect of *V*_G_ and visual signal**

Compared with synaptic behavior of artificial synapse under *V*_G_ = 0 V, similar synaptic behavior and larger -ΔPSCs are obtained with negative *V*_G_ applied to the device (*V*_G_ = -40 V).


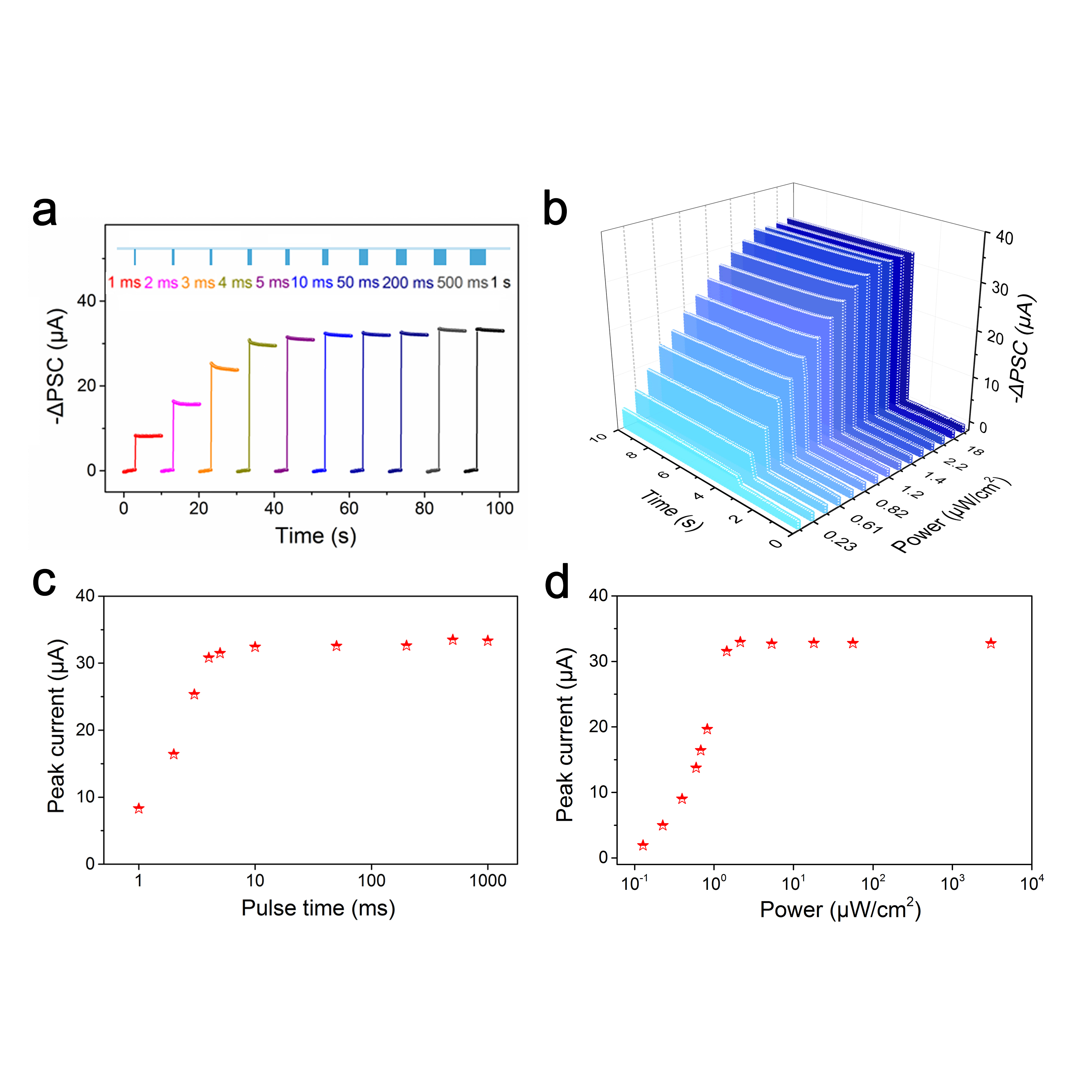


**Figure S14.** **Synergistic effect of *V*_G_ and visual signal for artificial photonic synapse.** **a** -ΔPSCs under different light pulse width (increase from 1 ms to 1 s) at a fixed *P*_light_ of 5.32 μW cm^-2^, *V*_G_ = -40 V and *V*_D_ = 0.5 V. **b** -ΔPSCs under different *P*_light_ (light pulse width, 5­0 ms; *V*_G_ = -40 V; *V*_D_ = 0.5 V). **c, d** The peak current of -ΔPSCs as a function of light pulse width and *P*_light_, respectively.

1. **-ΔPSC of the synaptic device under consecutive light pulses at different *V*_G_**

When 100 consecutive light pulses (*P*_light_ = 5.32 μW cm^-2^, light pulse width: 50 ms, *V*_G_ = 0 V) are applied to the synapse device at *V*_G_ = 0 V, the amplitude value of -ΔPSC linearly increases to 13 μA and tends to be stabilized at 16 μA. The amplitude rate of -ΔPSC, which is defined as A_n_/A_1_ (A is the amplitude of the -ΔPSC peak value), can reach ~350% and ~800% under *V*_G_ = 0 V and *V*_G_ = -40 V after the stimulation of consecutive light pulses for 100 times, respectively. Another key parameter determining the accuracy of image recognition: *G*_max_/*G*_min_ (defined as the ratio between the maximum and minimum conductance value) is also extracted in Fig. S15d, which increases from 132 to 804 with *V*_G_ increasing from 0 V to -40 V.


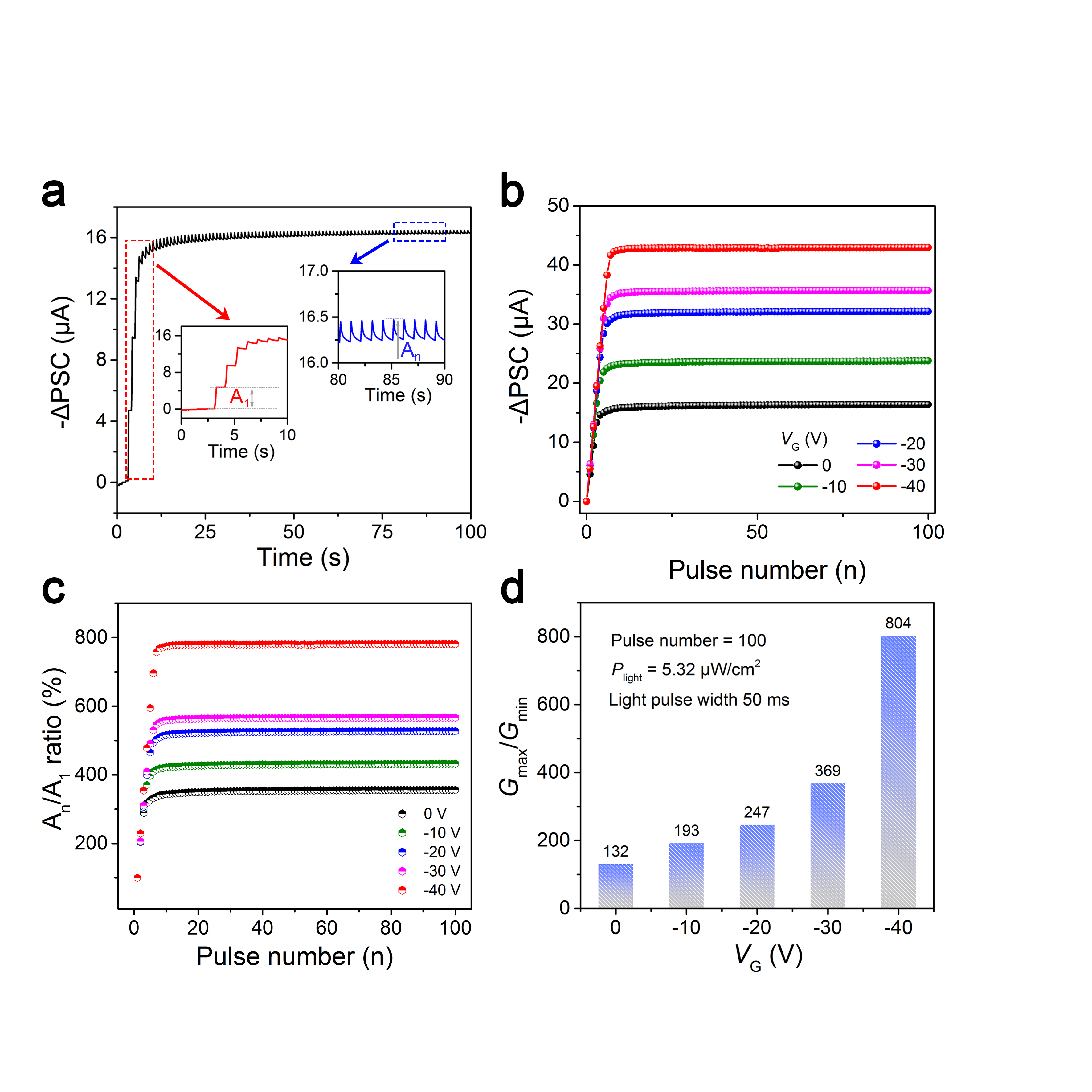


**Figure S15.** **-ΔPSC under consecutive light pulses at different *V*_G_. a** -ΔPSC under 100 consecutive light pulses (*P*_light_ = 5.32 μW cm^-2^, light pulse width: 50 ms, *V*_G_ = 0 V). Inset: the enlarged first -ΔPSC peak (A_1_, left) and last -ΔPSC peak (A_n_, right). **b** The -ΔPSCs as a function of pulse number with different *V*_G_ applied on Si. **c** The amplitude rate (A_n_/A_1_) as a function of pulse number with different *V*_G_ applied on Si. **d** The conductance margins (*G*_max_/*G*_min_) as a function of *V*_G_.

1. **-ΔPSC characteristics of the artificial photonic synapse under lower light intensity and shorter pulse time**

The -ΔPSC characteristics of this device show the same trend of change but larger amplitude rate (reach 2500% after the stimulation of consecutive light pulses) and good linear relationship under lower light intensity and shorter pulse time.


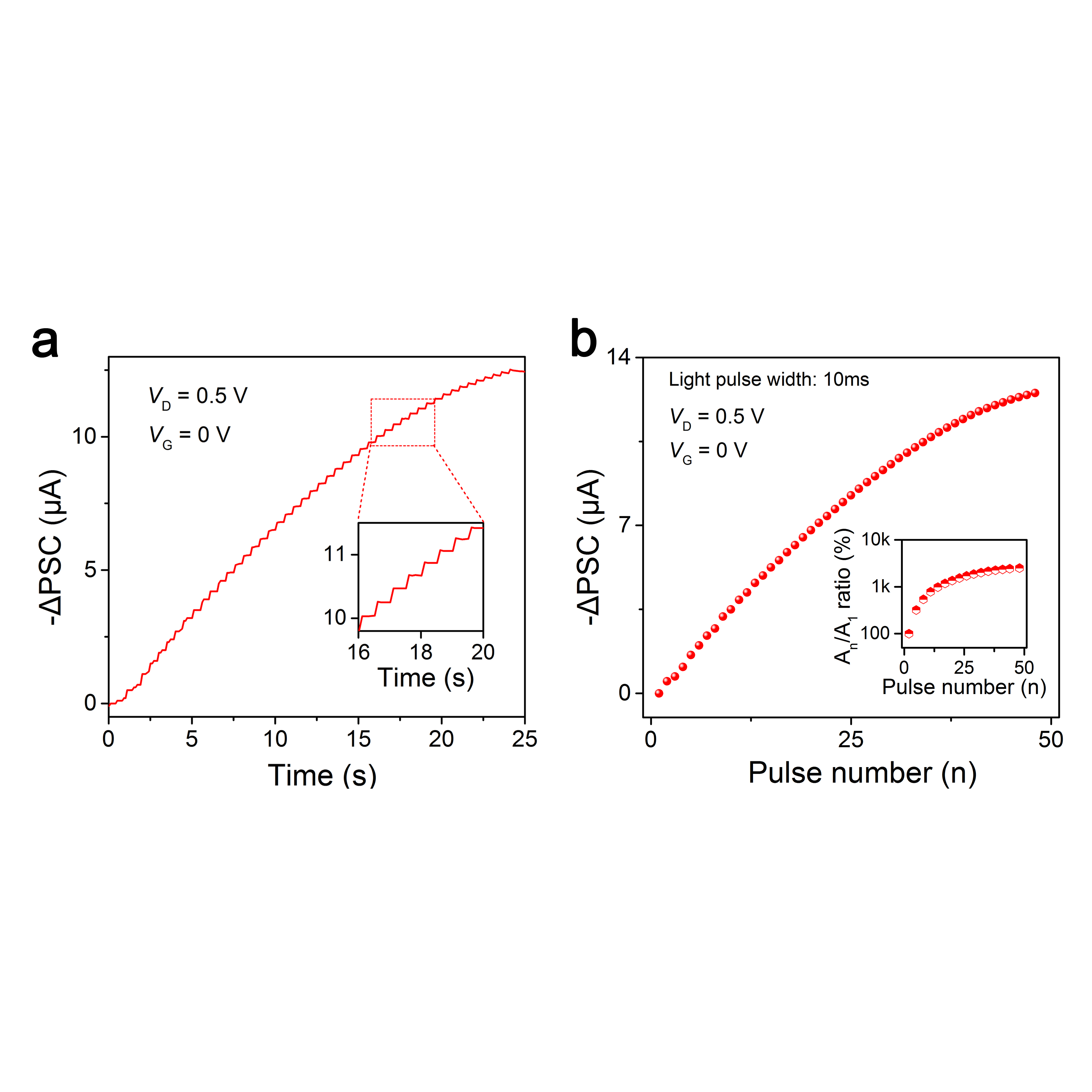


**Figure S16.** **-ΔPSC characteristics under lower light intensity and shorter pulse time.** **a** The -ΔPSC under consecutive light pulses illumination as a function of time. The *P*_light_ and light pulse width are fixed at 0.29 μW cm^-2^ and 10 ms, respectively. **b** The -ΔPSC as a function of pulse number with *P*_light_ at 0.29 μW cm^-2^. Inset: the calculated amplitude rate (A_n_/A_1_).

1. **Depression/potentiation (D/P) curve of synaptic device on SiO_2_/Si substrate**

The results indicate that -ΔPSC of the synaptic device shows an increasement tendency with the increased light intensity, and LTP/LTD curve shows the best linear relationship when *P*_light_ is fixed at 0.29 μW cm^-2^, which is beneficial to the improvement of accuracy of fully-connected optoelectronic neural network (FONN) simulation.


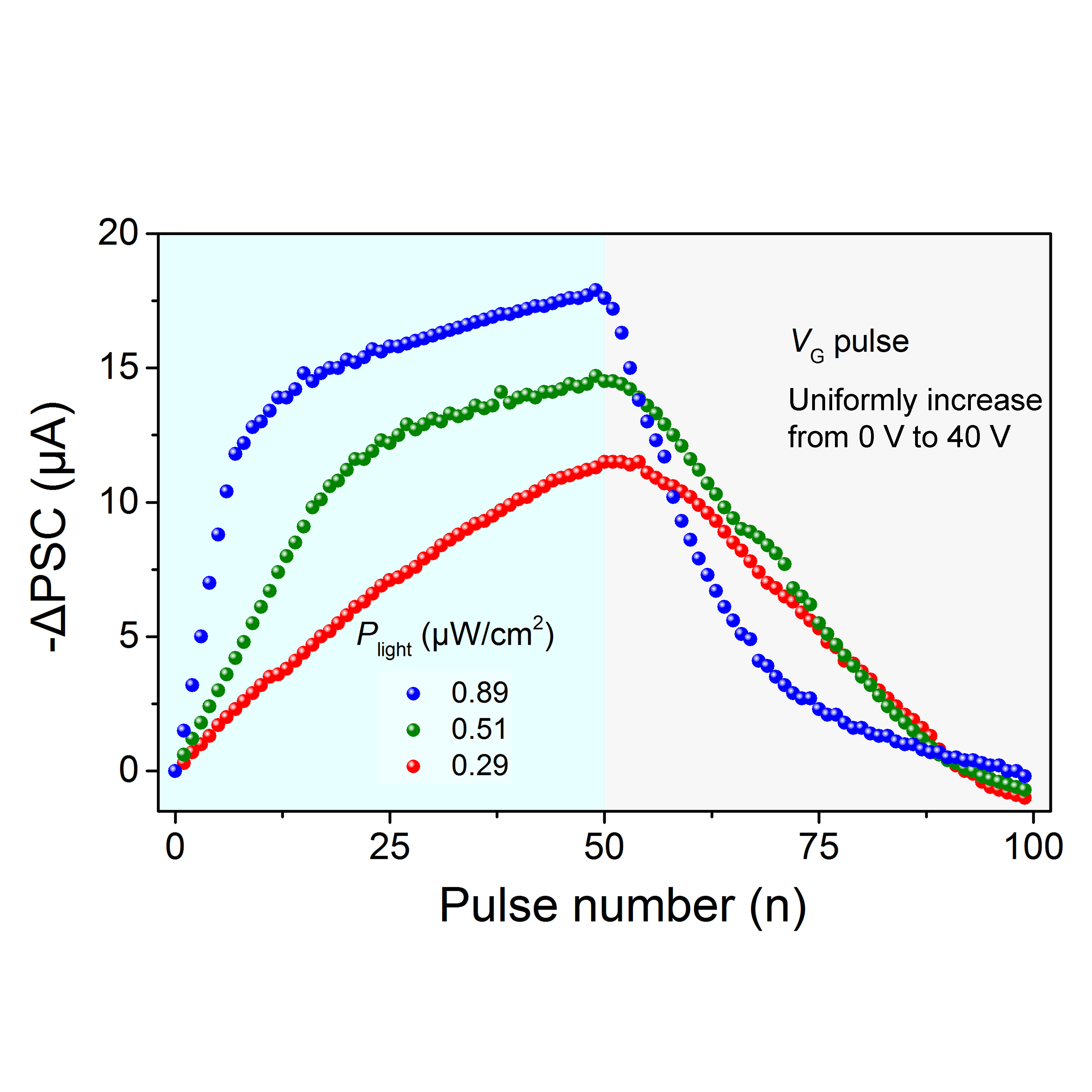


**Figure S17.** LTP/LTD characteristics trained by 50 consecutive potentiation pulses (optical programming) and depression pulses (electrical erasing) under different light intensity on SiO_2_/Si substrate.

1. **Endurance characteristics of the synaptic device**

The pulse-switching characteristics of the synaptic device is shown in Fig. S18a, where the device is periodically switched between program and erase state with more than 150 cycles over 3000 s. The result exhibits that the device can be switched well between the program and erase state, indicating good endurance performance. Furthermore, endurance performance of potentiation-depression is shown in Fig. S18b, the potentiation and depression processes can be continuously simulated by applying light pulses and *V*_G_ spikes (200 points for each cycle), reflecting repeatable switching and good endurance of the device.


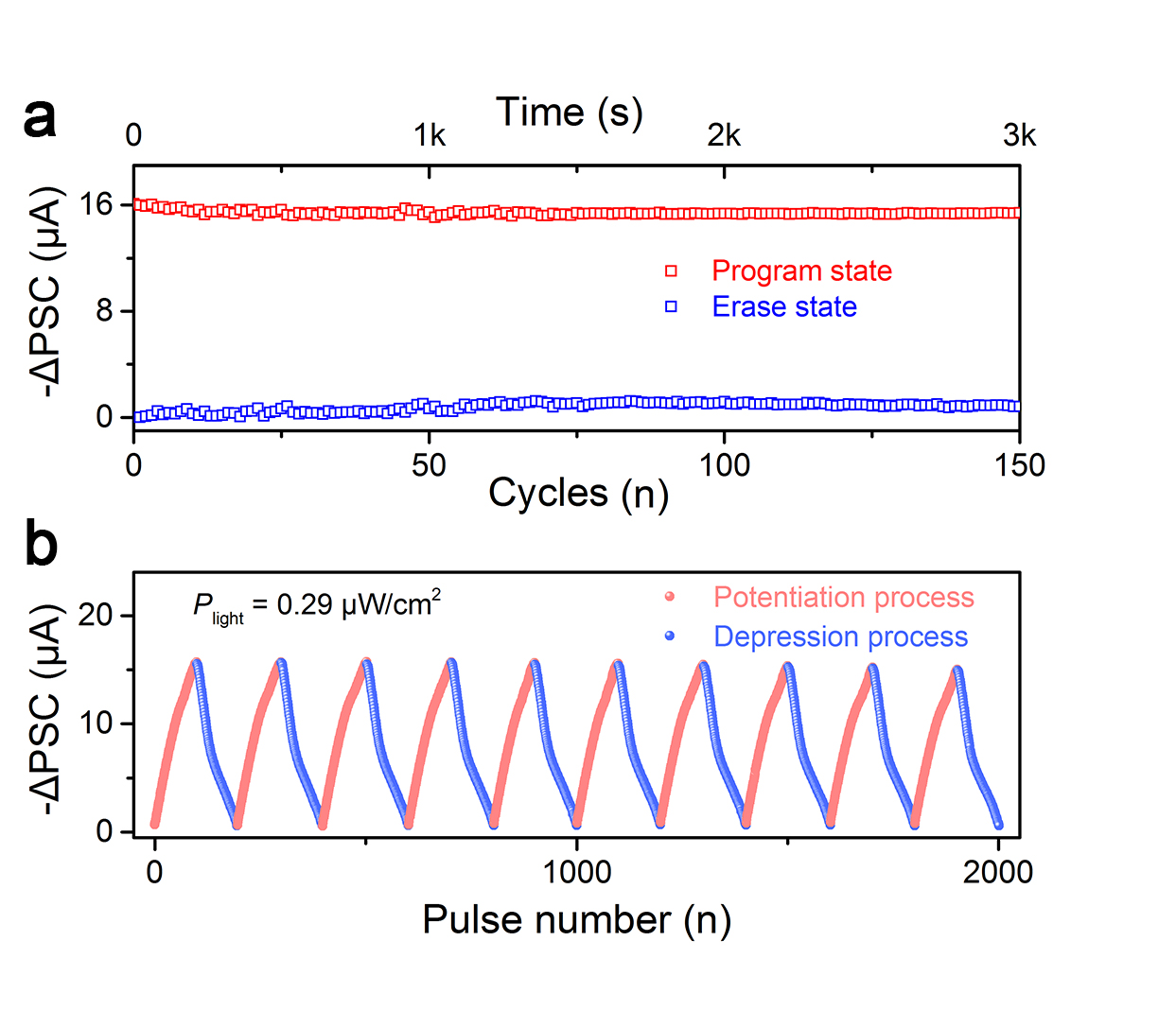


**Figure S18. Endurance characteristics of the device.** **a** Pulse-switching characteristic of the synaptic device. The programming process is initiated by a light pulse (450 nm) with a power intensity of 125.7 μW cm^-2^ and a pulse width of 2 s, and the erasing process is initiated by an electrical pulse (*V*_G_ = 40 V; pulse width: 2 s). **b** Endurance performance of potentiation-depression with 2000 consecutive spikes. Ten cycles with one hundred distinct states are shown for potentiation and depression processes, respectively, which are triggered by repeated light pulse and positive gate voltage.

1. **Synaptic characteristics of a graphene/h-BN/BP-CdS device**

The synaptic device with graphene as gate and boron nitride as insulating layer shows similar synaptic properties as the device on SiO_2_/Si substrate but lower gate voltage, further proving that the synaptic behavior originates from the heterostructure rather than substrate.


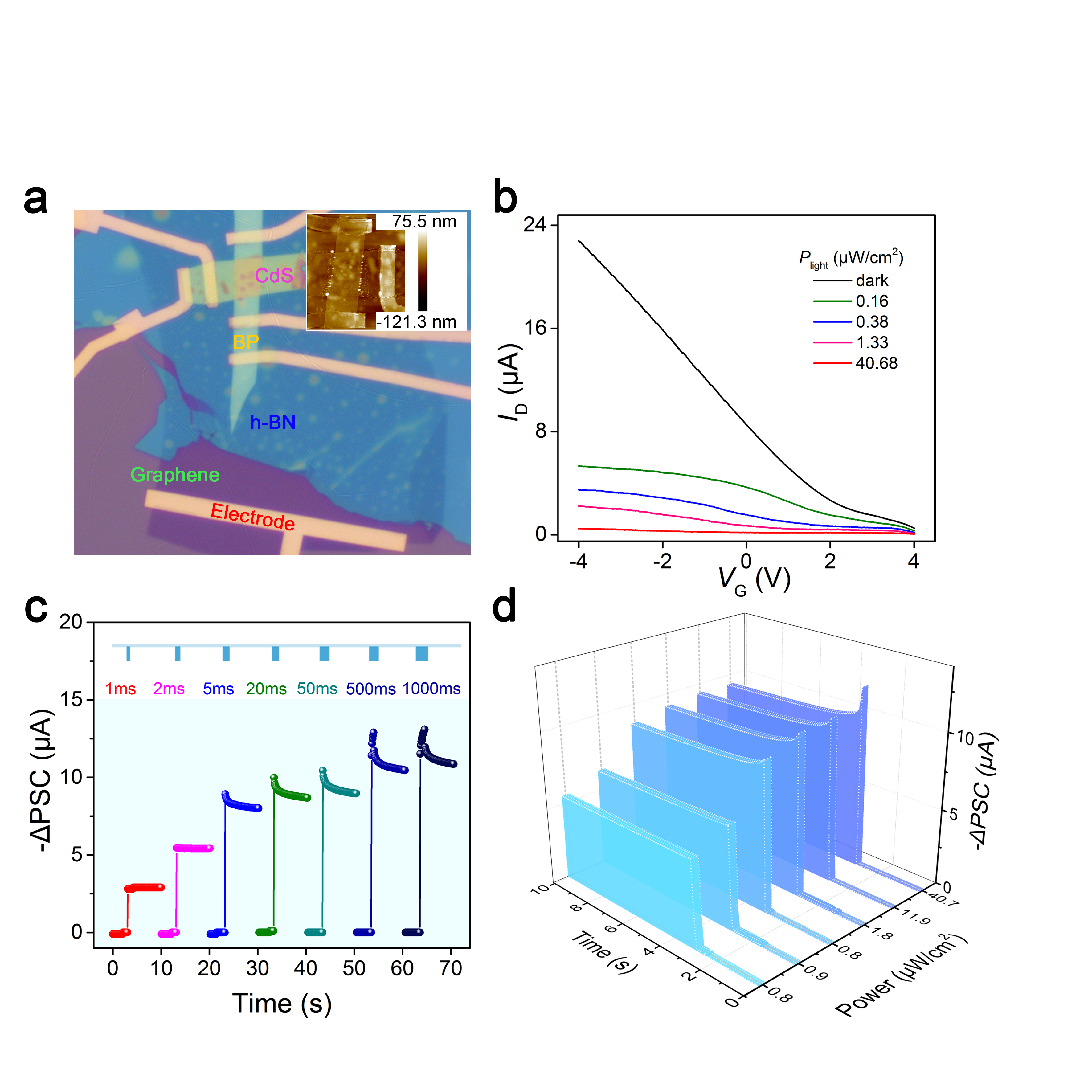


**Figure S19.** **Synaptic characteristics of the device with graphene as gate and** **boron nitride as insulating layer.** **a** The optical and AFM image of the constructed device. BP: ~15 nm; CdS: ~20 nm; h-BN: ~16 nm; Graphene: ~4 nm. **b** Transfer curves of HJ-FET in dark and under different light power illumination. **c** -ΔPSCs under different light pulse width (from 1 ms to 1 s) at a fixed *P*_light_ of 11.97 μW cm^-2^, *V*_G_ = 0 V and *V*_D_ = 0.5 V. **d** -ΔPSCs under different *P*_light_ (light pulse width, 50 ms; *V*_G_ = 0 V; *V*_D_ = 0.5 V).

**Supplementary Note 1**

**Calculation and extraction** **of energy consumption for the synaptic device**

The estimation of energy consumption for the synaptic device is based on the following equations:

$E_{\mathrm{programming}}=P_{\mathrm{spike}}\times A_{\mathrm{active}}\times T_{\mathrm{duration}}$ (2)

$E_{\mathrm{erasing}}=V_{G}\times I_{G}\times T_{\mathrm{duration}}$ (3)

Here, *E*_programming_ and *E*_erasing_ denote the energy consumption in single photonic programming process and electric erasing process, respectively. *P*_spike_, *A*_active_ and *T*_duration_ are the optical spike power, the active channel area and the spike duration, respectively^8^. *V*_G_ and *I*_G_ represent gate voltage and gate leakage current.

**References**

1. Li, D. et al. Light-triggered two-dimensional lateral homogeneous p-n diodes for opto-electrical interconnection circuits. *Science Bulletin* **65**, 293-299 (2020).
2. Liu, H. et al. Polar-induced selective epitaxial growth of multijunction nanoribbons for high-performance optoelectronics. *ACS Applied Materials & Interfaces* **11**, 15813-15820 (2019).
3. Li, D., Wang, B., Chen, M., Zhou, J. & Zhang, Z. Gate-controlled BP-WSe_2_ heterojunction diode for logic rectifiers and logic optoelectronics. *Small* **13**, 1603726 (2017).
4. Li, D., Chen, M., Zong, Q. & Zhang, Z. Floating-gate manipulated graphene-black phosphorus heterojunction for nonvolatile ambipolar schottky junction memories, memory inverter circuits, and logic rectifiers. *Nano Letters*. **17**, 6353-6359 (2017).
5. Li, L. et al. Black phosphorus field-effect transistors. *Nature Nanotechnology* **9**, 372-377 (2014).
6. Yu, J. et al. Bioinspired mechano-photonic artificial synapse based on graphene/MoS_2_ heterostructure. *Science Advances* **7**, eabd9117 (2021).
7. Wang, S. et al. A MoS_2_/PTCDA hybrid heterojunction synapse with efficient photoelectric dual modulation and versatility. *Advanced Materials* **31**, 1806227 (2019).
8. Seo, S. et al. An optogenetics-inspired flexible van der Waals optoelectronic synapse and its application to a convolutional neural network. *Advanced Materials* **33**, 2102980 (2021).
